# The adipokine leptin modulates adventitial pericyte functions by autocrine and paracrine signalling

**DOI:** 10.1038/s41598-017-05868-y

**Published:** 2017-07-14

**Authors:** Federica Riu, Sadie C. Slater, Eva Jover Garcia, Iker Rodriguez-Arabaolaza, Valeria Alvino, Elisa Avolio, Giuseppe Mangialardi, Andrea Cordaro, Simon Satchell, Carlo Zebele, Andrea Caporali, Gianni Angelini, Paolo Madeddu

**Affiliations:** 1 Bristol Heart Institute, School of Clinical Sciences, University of Bristol, Bristol Royal Infirmary, Bristol, BS2 8HW United Kingdom; 2 University of Nottingham, Cancer Biology, Division of Cancer and Stem Cells, School of Medicine University of Nottingham, Nottingham, NG7 2UH United Kingdom; 3 Centre for Cardiovascular Science, Queen’s Medical Research Institute, Edinburgh, EH16 4TJ United Kingdom

**Keywords:** Biological techniques, Stem-cell research

## Abstract

Transplantation of adventitial pericytes (APCs) improves recovery from tissue ischemia in preclinical animal models by still unknown mechanisms. This study investigates the role of the adipokine leptin (LEP) in the regulation of human APC biological functions. Transcriptomic analysis of APCs showed components of the *LEP* signalling pathway are modulated by hypoxia. Kinetic studies indicate cultured APCs release high amounts of immunoreactive LEP following exposure to hypoxia, continuing upon return to normoxia. Secreted LEP activates an autocrine/paracrine loop through binding to the LEP receptor (LEPR) and induction of STAT3 phosphorylation. Titration studies using recombinant LEP and siRNA knockdown of *LEP* or *LEPR* demonstrate the adipokine exerts important regulatory roles in APC growth, survival, migration and promotion of endothelial network formation. Heterogeneity in LEP expression and secretion may influence the reparative proficiency of APC therapy. Accordingly, the levels of LEP secretion predict the microvascular outcome of APCs transplantation in a mouse limb ischemia model. Moreover, we found that the expression of the *Lepr* gene is upregulated on resident vascular cells from murine ischemic muscles, thus providing a permissive milieu to transplanted LEP-expressing APCs. Results highlight a new mechanism responsible for APC adaptation to hypoxia and instrumental to vascular repair.

## Introduction

Multipotent mesenchymal cells from the vascular wall, alias adventitial pericytes (APCs), are emerging as promising candidates for therapeutic vasculogenesis^1–4^. Evidence from our group and others indicate that transplantation of human APCs promotes vascular and muscular repair in models of peripheral and myocardial ischemia^5–9^. Transplanted APCs incorporate into the host’s vasculature and release angiocrine factors, including Angiopoietin-1, VEGF-A and microRNA-132. The angiocrine activity of APCs is enhanced by hypoxia, thus accounting for their advantaged proangiogenic action upon transplantation into an ischemic tissue^9, 10^. However, paracrine mechanisms activated by hypoxia are still incompletely understood.

Leptin (LEP) is a hypoxia-inducible adipocytokine that plays a key role in the central regulation of food intake and energy expenditure^11–13^, and also participates in the modulation of immunity^14^, bone formation^15^, and cardiovascular function^16^. Seminal evidence indicates components of the LEP pathway are expressed in human stem cells, but the pathophysiological implications of the LEP signalling remain unclear and controversial. The LEP receptor (LEPR) is highly expressed by embryonic stem cells and induced pluripotent stem cells, suggesting that LEP regulates relevant functions in these primitive cells^17^. In the adult bone marrow (BM), the LEPR identifies medullary mesenchymal stem cells (MSCs)^18^ as well as pericyte-like stromal cells that support hematopoiesis within the vascular niche^19–21^. Upregulation of the *LEPR* gene in cultured BM-MSCs has been associated with cessation of growth due to replicative senescence^22^. Two recent studies showed that hypoxia-preconditioned murine BM-MSCs exert enhanced regenerative actions through an amplification of the LEP signalling^23, 24^. Nevertheless, the functional relevance of the LEP pathway in human pericytes remains unknown.

The present study investigates the expressional regulation and functional actions of LEP in human APCs. We show for the first time that APCs secrete abundant levels of LEP under hypoxia, by one order of magnitude higher than BM-MSCs. APC-derived LEP exerts autocrine promotion of APCs survival and migration as well as paracrine stimulation of endothelial cell (EC) proliferation, permeability, and network formation. Importantly, the levels of secreted LEP predict the angiogenic activity of APC transplantation in a murine model of peripheral ischemia. Moreover, the expression of the *Lepr* gene is upregulated on vascular cells from mouse ischemic muscles, thus increasing resident cells sensitivity to the restorative action of LEP-releasing APCs. Altogether, these results revealing a novel signalling mechanism instrumental to APC functionalities may have a far-reaching and critical impact on cardiovascular regenerative medicine.

## Results

### Effect of hypoxia on global gene expression in human APCs

Saphenous vein leftovers were obtained from 24 patients undergoing coronary artery bypass graft surgery. We succeeded in isolating and expanding 21 APC lines (88% of processed veins), using a standard protocol described in the methods section and previous publications^5, 6^. Clinical and demographic characteristics of the corresponding 21 patients are provided in Supplementary Table I. The phenotype of human APCs have been extensively characterized^2, 5, 6, 9^. In four biological replicates, here we demonstrate APCs have similar morphology when cultured under normoxia or hypoxia, as assessed by contrast phase microscopy (Supplementary Figure Ia). Likewise, hypoxia does not alter the expression of surface markers, as identified by flow cytometry and immunocytochemistry (Supplementary Figure Ib–e and Supplementary Table II).

**Figure 1:**
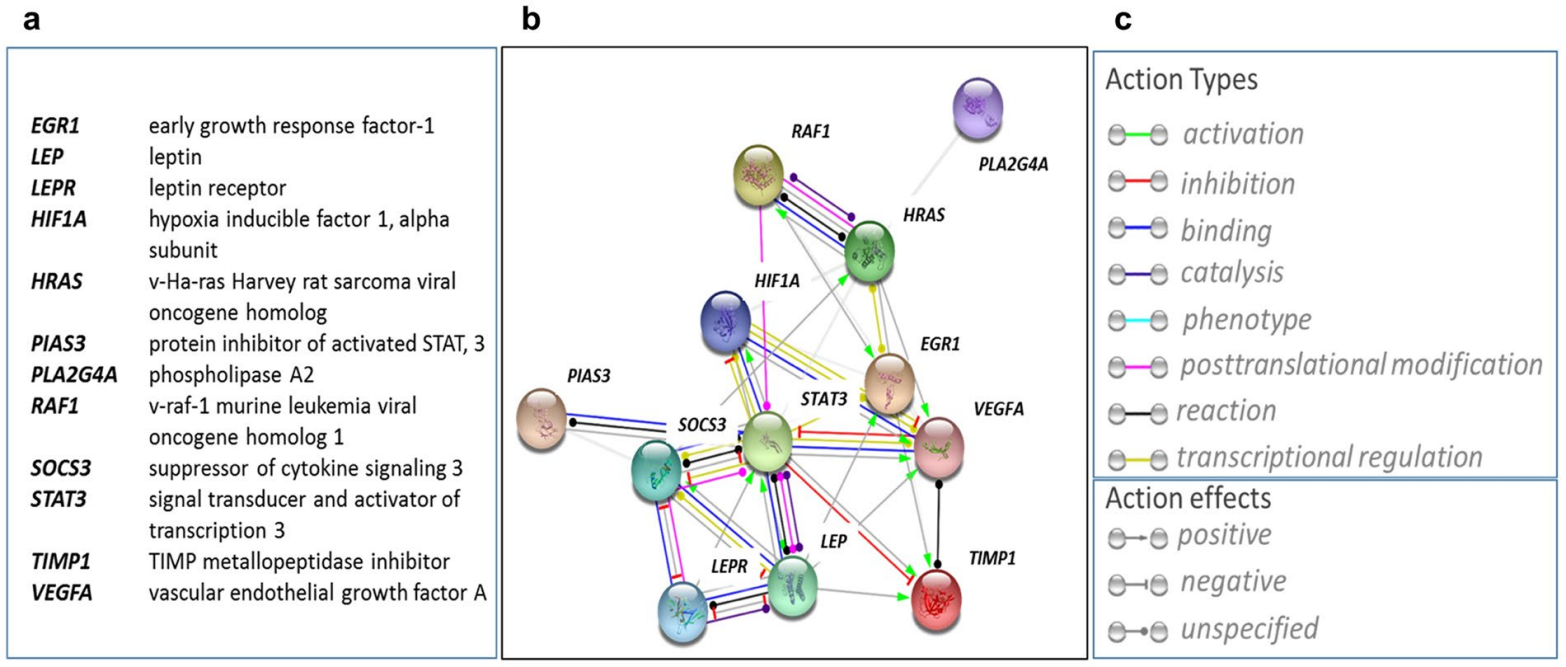
Hypoxia modulates the *LEP* signalling pathway in human APCs. Transcriptional network generated by the String software on data derived from the Agilent array. (**a**) Legend of the impacted genes, (**b**) graphical illustration of the interconnected genes, and (**c**) legend of the type and effect of actions illustrated by interconnecting lines.

Results from an Agilent human gene microarray on four individual APC lines, selected at random from the collective of 21 cell lines, indicate that 410 out of 2050 expressed genes are modulated by hypoxia, with a log2 fold change >1 and a *p*-value < 0.05. Here, in the interest of conciseness and clarity, we report the list of the top 131 genes that exhibited a highly-significant (*p* < 0.01) upregulation (n = 72) or downregulation (n = 59) following hypoxia Supplementary Table III. In particular, *LEP* was found to be strongly upregulated by hypoxia (log2 fold change: 1.83, *p* = 0.006) together with several members of the *LEP* signalling pathway (Fig. 1). Statistical analysis using the String software available at http://string-db.org/ indicates a biologically relevant interaction among the studied genes (enrichment *p*-value = 1.11e-16). In addition, using the Ingenuity Pathway Analysis (IPA) software, we found that *LEP* is associated with all the main biofunctions/pathways found to be significantly modulated by hypoxia, including the regulation of cell death and survival, development, energy production and tissue morphology (Supplementary Figure II). Next, by screening the networks encompassing the highest number of genes regulated in the dataset (Supplementary Table IV), we found that *LEP* is modulated together with other 18 genes in a network of 29 genes, whose function is associated with cell death/apoptosis and cell-to-cell signalling/interaction (Supplementary Table V). To the best of our knowledge, this is the first report on the global gene signature induced by low oxygen in human APCs.

### Human APCs produce and release remarkable amounts of LEP under hypoxia

**Figure 2:**
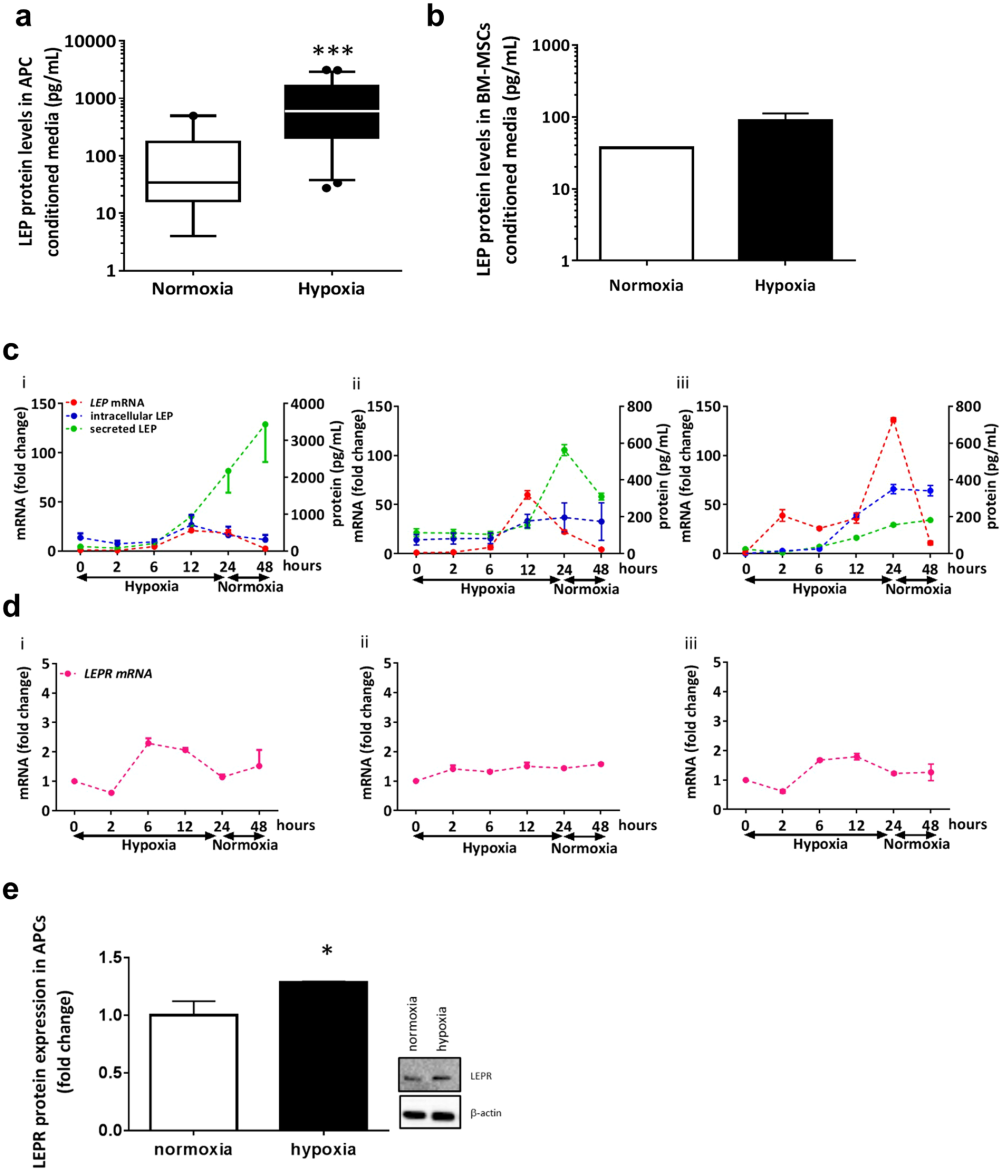
Hypoxia induces leptin production and secretion by human APCs. (**a**) Highly induced LEP protein levels in APC conditioned media collected under hypoxia as compared with normoxia. Data are medians and 5–95 percentiles from twenty-one APCs lines. ***p < 0.01. (**b**) LEP protein levels in conditioned media of BM-MNCs (n = 3, *p* = 0.09 *vs*. normoxia). (**c**) Time course of *LEP* transcription and LEP protein production and secretion under hypoxia and following return to normoxia in three individual APC lines. Data are mean and SEM. (**d**) Concomitant changes in *LEPR* mRNA levels in the same cell lines. (**e**) Representative immunoblots for the expression of LEPR protein in APCs under normoxia and hypoxia during 48 h. Band densitometry was determined using Image J software and the fold change was calculated *per* each APC line (n = 3). Data are shown as mean ± SEM and asterisk means statistical significance as per *p* = 0.049.

We next assessed the capacity of human APCs to secrete LEP. Measurement of LEP protein levels in conditioned media by ELISA showed a marked increase following 48 h exposure to hypoxia (Fig. 2a, n = 21, *p* < 0.001 *vs*. normoxia). A reference analysis of the secretory ability of BM-MSCs, collected from patients undergoing hip replacement surgery (see methods for details), showed no significant change in LEP levels following hypoxia (Fig. 2b, n = 3, *p* = 0.09 *vs*. normoxia). No association was found when comparing LEP secretion and subjects’ age, body mass index, smoking habit and grading of angina pectoris.

To gain an accurate insight into the kinetics of the LEP signalling induction, we studied the time course of *LEP* and *LEPR* transcription and LEP intracellular content and secretion in three individual APCs lines. Results show *LEP* mRNA levels peak between 12 and 24 h of hypoxia, with 24 h post-hypoxic normoxia being sufficient for restoration of the basal expressional state. In contrast, intracellular and secreted LEP protein levels remained elevated even after normoxia restoration (Fig. 2c). The expression of *LEPR* was variable but not significantly changed during exposure to hypoxia and return to normoxia (Fig. 2d). LEPR protein, assessed by Western blot, was upregulated by 1.29 ± 0.01 fold change (*p* < 0.05) in APCs exposed to hypoxia for 48 h (Fig. 2e), the time point chosen in our previous experiments.

It is known that *LEP* is transcriptionally regulated by the Hypoxia-Inducible Factor 1-alpha (HIF1α) through binding of the transcription factor to the hypoxia response element (HRE) promoter site of the *LEP* gene.

In addition, a recent investigation showed that forced miR-210 expression remarkably increases LEP secretion in murine HL-1 cardiomyocytes^25^. Also, in hypothalamic neurons and NIH/3T3 reporter cells, miR-210 enhances LEP signalling by inhibiting the protein-tyrosine phosphatase PTP1B, which acts as a negative regulator through the dephosphorylation of the LEPR-associated Janus kinase 2 (JAK2)^25, 26^. Among the differentially expressed microRNAs, the Agilent array identified miR-210 to be strongly upregulated in human APCs exposed to hypoxia. This data was confirmed by qPCR analysis, showing a 14-fold increase in miR-210 levels after 48 h exposure to hypoxia (Supplementary Figure IIIa). We next inhibited miR-210 in four human APC lines using a specific antago-miR. Effective inhibition of miR-210 was confirmed by qPCR, either in normoxia or hypoxia (Supplementary Figure IIIa). However, the *LEP* gene and intracellular or secreted LEP protein expression were not affected by the miR-210 inhibition (Supplementary Figure IIIb–d). When considering PTP1B, we found no change under hypoxia *vs*. normoxia (Supplementary Figure IIIe), and observed a modest but significant upregulation following miR-210 inhibition in hypoxic APCs (*p* < 0.05 *vs*. scramble, Supplementary Figure IIIf). However, this effect was associated with no change in the phosphorylation levels of JAK2 (Supplementary Figure IIIg). Altogether, these data militate against a role of hypoximiR-210 in the modulation of the LEP signalling, which is at variance with the results of previous studies in other cell types^25, 26^.

### Human APCs are functionally responsive to exogenous LEP stimulation

**Figure 3:**
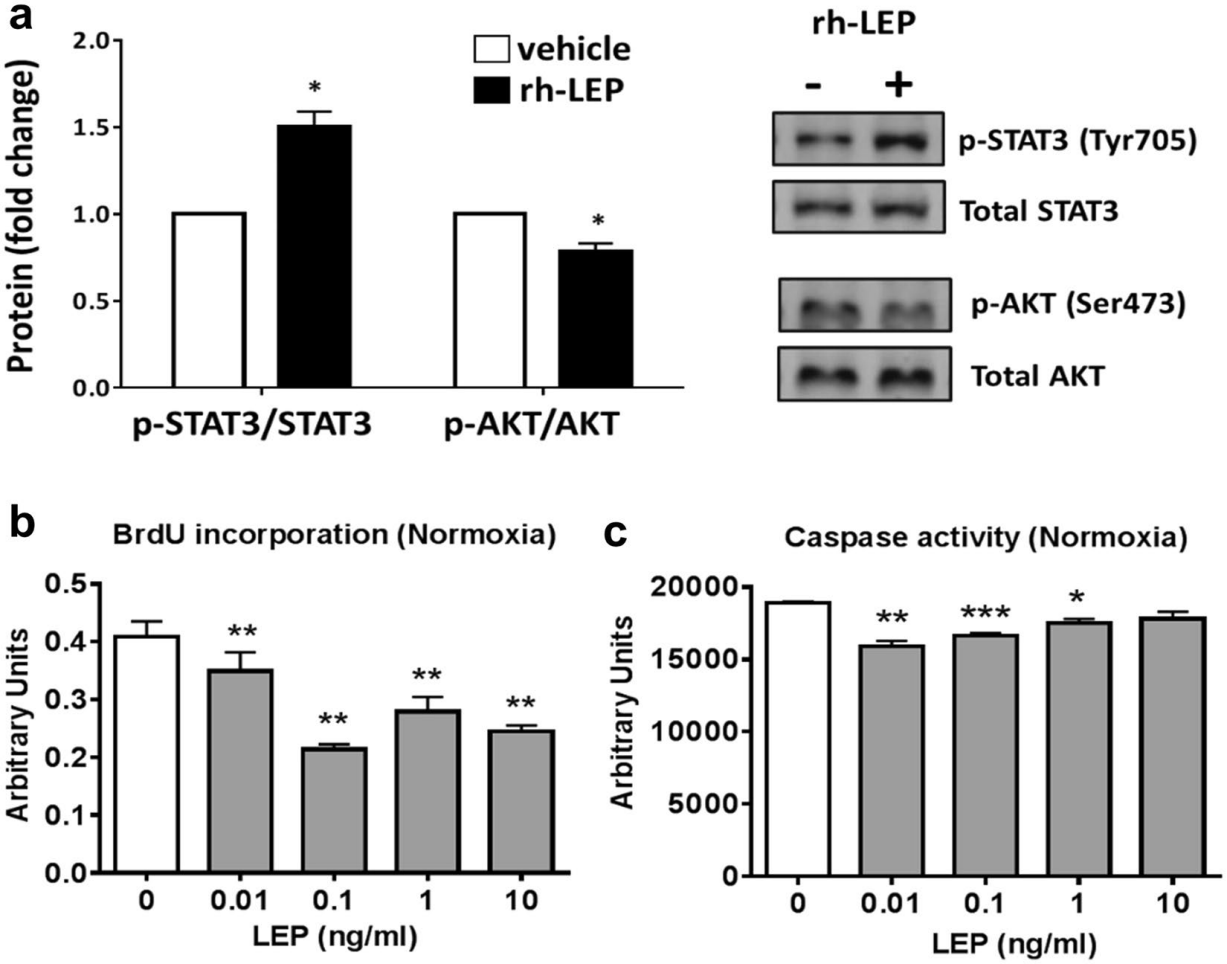
Effect of exogenous recombinant leptin on canonical signalling and functional assays in human APCs. (**a**) Representative immunoblots of phosphorylated and total proteins. Bar graphs show the average of four biological replicates. Cells were stimulated with 1 ng/mL rh-LEP. *p < 0.05 *vs*. vehicle. (**b**) Proliferation assay of four APC lines performed following stimulation with increasing doses of rh-LEP under normoxia. *p < 0.05 and **p < 0.01 *vs*. vehicle. (**c**) Apoptosis assay performed under normoxia. *p < 0.05, **p < 0.01 and ***p < 0.001 *vs*. vehicle. Values are mean and SEM.

We next assessed the responses evoked by recombinant human LEP (rhLEP) on human APCs under normoxic conditions. The molecular analysis focused on STAT3 and AKT, which are canonical mediators of LEP intracellular signalling. In four APCs lines tested, rhLEP significantly increased the phosphorylation of STAT3 (Tyr105) while reducing AKT phosphorylation (*p* < 0.05 for both comparisons, Fig. 3a). Moreover, we explored the functional effects of increasing doses of rhLEP up to the upregulated endogenous levels seen under hypoxia. Interestingly, exposure of APCs to rhLEP reduces proliferation (assessed by BrdU incorporation, Fig. 3b) and exerts anti-apoptotic actions (assessed by caspase 3/7) (Fig. 3c). The anti-proliferative response was dose-related while the anti-apoptotic effect reached a plateau already at the lowest rhLEP dosage. These data highlight the functional competence of the LEP signalling machinery in human APCs.

### Autocrine action of endogenous LEP in human APCs

**Figure 4:**
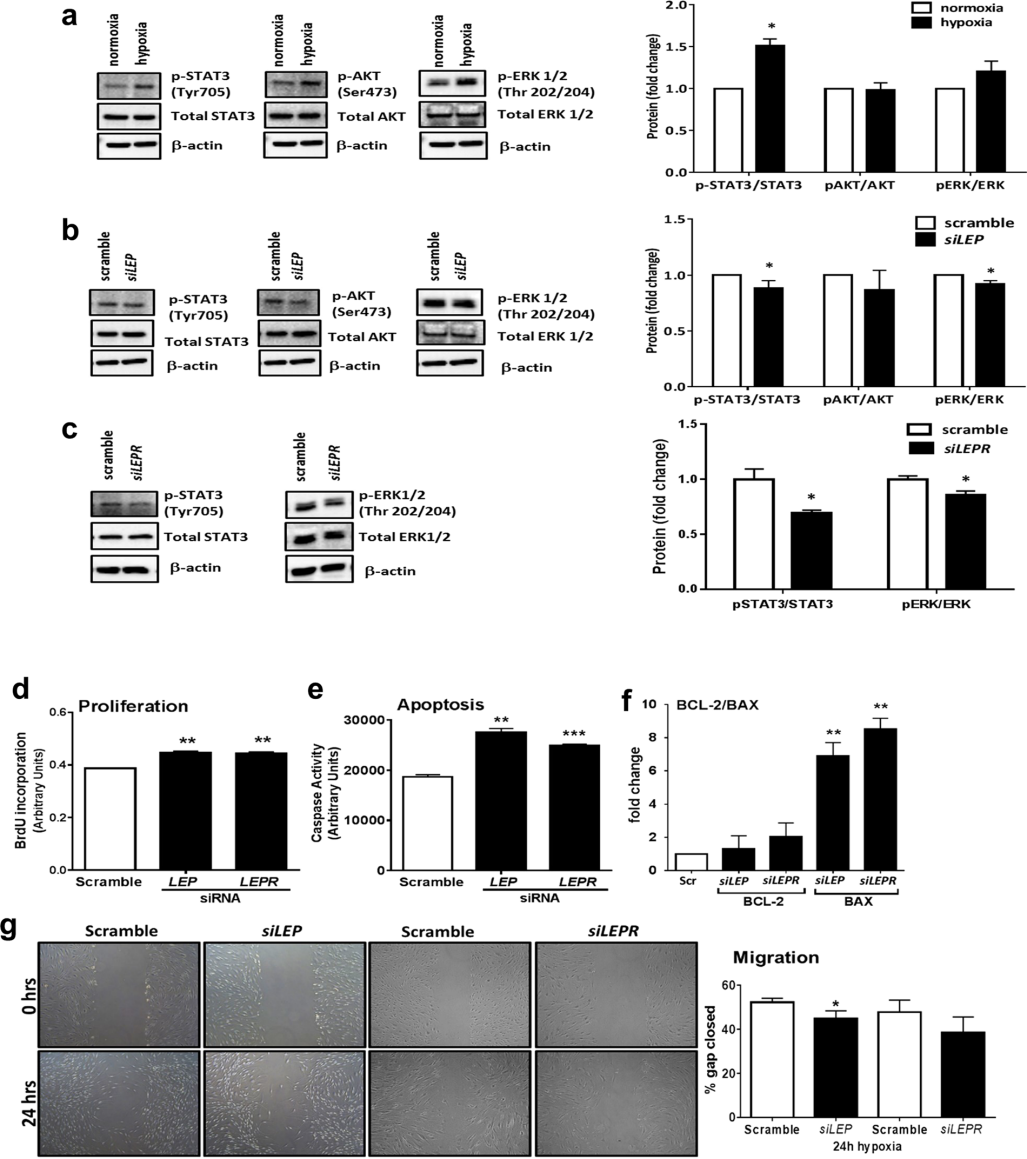
Effect of LEP silencing. Expression and phosphorylation levels of leptin-associated kinases in normoxic and hypoxic APCs (**a**), and effect of *LEP* (**b**) or *LEPR* (**c**) silencing on kinase phosphorylation in hypoxic APCs. Representative immunoblots of phosphorylated and total proteins are displayed. Bar graphs show the average of four biological replicates. *p < 0.05 *vs*. normoxia. (**d**–**f**) Silencing of *LEP* and its receptor in hypoxic APCs induces cell proliferation (**d**) and also exerts pro-apoptotic effects as assessed by the caspase activity assay (**e**) and the expression of BCL-2 and BAX (**f**). **p < 0.01 and ***p < 0.001 *vs*. scramble. (**g**) *LEP* silencing reduces the migratory activity of APCs in a scratch assay performed under hypoxia, however *LEPR* silencing was unable to induce significant changes in APC migration. Representative contrast microscopy images and bar graph showing average data. *p < 0.05 *vs*. scramble.

Having demonstrated that human APCs are responsive to an artificial increase in the environmental levels of LEP, we next investigated the functional relevance of the autocrine LEP signalling stimulated by hypoxia. First, we confirmed that hypoxia induces the phosphorylation of STAT3 in human APCs. There was no significant change in pERK and pAKT levels before and after hypoxia (Fig. 4a). We then silenced *LEP* or *LEPR* by siRNA in four hypoxic APC lines, using scramble sequences as a control. The effective knockdown was confirmed at mRNA and protein level (Supplementary Figure IVa–c). Importantly, *LEP* silencing reduced STAT3 and ERK phosphorylation in hypoxic APCs (Fig. 4b). Silencing of the *LEPR* reproduced the same effect in hypoxic APCs (Fig. 4c). STAT3 and ERK are canonical downstream modulators of LEP biological actions; therefore, their reduction after *LEP* or *LEPR* silencing confirm that hypoxia activates a canonical kinase signalling in APCs. Next, we explored the effect of LEP inhibition on hypoxic APCs. Results indicate that knockdown of *LEP* results in increased cell proliferation (Fig. 4d) and apoptosis (Fig. 4e and f). Similar results were observed following silencing of the *LEPR* (Fig. 4d–f), thus suggesting an autocrine loop of secreted LEP. Moreover, *LEP* silencing significantly decreased the migration of hypoxic APCs in an *in vitro* scratch assay, however silencing of the *LEPR* produced no significant effect on migration (Fig. 4g).

### Secreted LEP contributes to APC-induced promotion of angiogenesis

**Figure 5:**
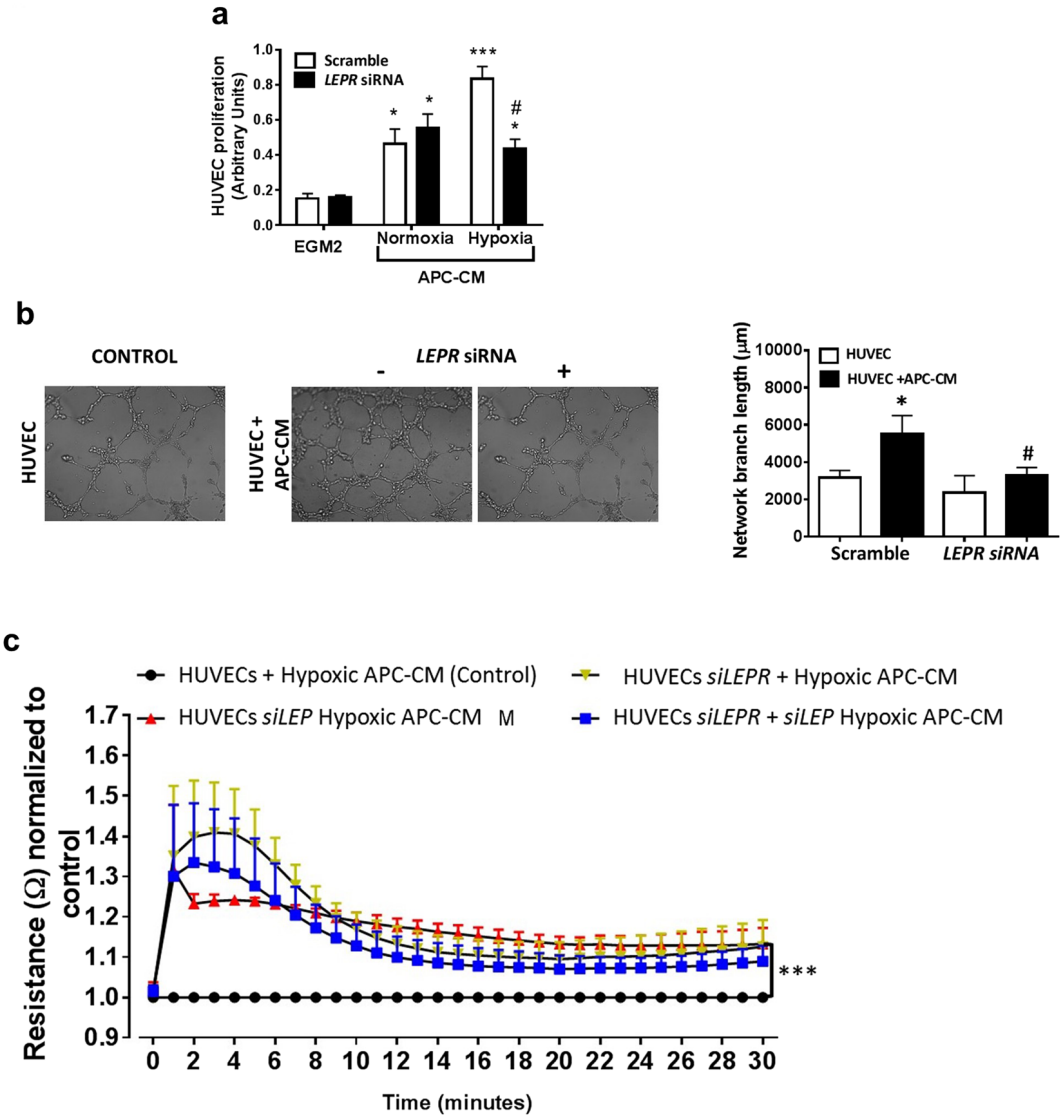
Angiocrine activity of APC-secreted LEP (**a**) *LEPR* silencing inhibited the proliferative effect of hypoxic APC-CM on HUVECs. *p < 0.05 and ***p < 0.001 *vs*. EGM2 ^#^ *p* < 0.05 *vs*. scramble. (**b**) *LEPR* silencing abolished the promotion of HUVEC network formation by hypoxic APC-CM. *p < 0.05 *vs*. EGM2 ^#^ *p* < 0.05 *vs*. scramble. (**c**) Blockade of *LEP* signalling increases the vascular resistance of HUVEC monolayers exposed to hypoxic APC-CM. ***p < 0.001 *vs*. control.

Previous studies have shown that APCs interact with ECs to stimulate angiogenesis^5, 6, 9^, but it is not known if LEP contributes to this action. Hence, we next investigated the effect of APC-derived LEP on EC functions, including proliferation, network formation, and permeability. To this aim, *LEPR*-silenced or scramble-treated HUVECs were exposed to conditioned media collected from normoxic or hypoxic APCs. As expected, siRNA silencing significantly reduced the expression of the *LEPR* in HUVECs (Supplementary Figure IVd), which made them less responsive to the stimulatory action of hypoxic APC-CM on proliferation (Fig. 5a) and network formation (Fig. 5b). Initial stages of angiogenesis are associated with the loss of mutual contacts between ECs, resulting in increased permeability of the endothelial layer. We found silencing *LEP* in APCs or *LEPR* in HUVECs results in decreased ability of hypoxic APC conditioned media to induce endothelial permeability as assessed by evaluating HUVEC monolayer electrical resistance (Fig. 5c). Altogether, these data indicate that secreted LEP contributes to the functional cross-talk between APCs and ECs.

### LEP expression predicts the angiogenic effect of APC transplantation in a model of peripheral ischemia

Having demonstrated that APC-produced LEP promotes autocrine and angiocrine effects *in vitro*, we next investigated if the adipokine helps to predict the outcomes of APC therapy in a mouse model of limb ischemia. In line with the recommended use of historical data to reduce animal numbers (The National Centre for the Replacement Refinement & Reduction of Animals in Research, https://www.nc3rs.org.uk), we conducted a retrospective analysis of a published study from our laboratory^10^. The study consisted of *in vitro* functional and biochemical assays on five APC lines (including measurement of LEP levels in conditioned media collected under hypoxia) and *in vivo* experiments on inbred C57BL/6 mice, which were randomly assigned to vehicle or APC therapy (n = 7 *per* group). All mice completed the assessment of outcomes, i.e. blood flow recovery of the ischemic limb (laser Doppler) and muscular capillary density (immunohistochemistry), at the end of the 28 days follow up. Both *in vivo* outcomes and LEP measurements in APC conditioned media were assessed by investigators blind to the treatment randomization protocol.

**Figure 6:**
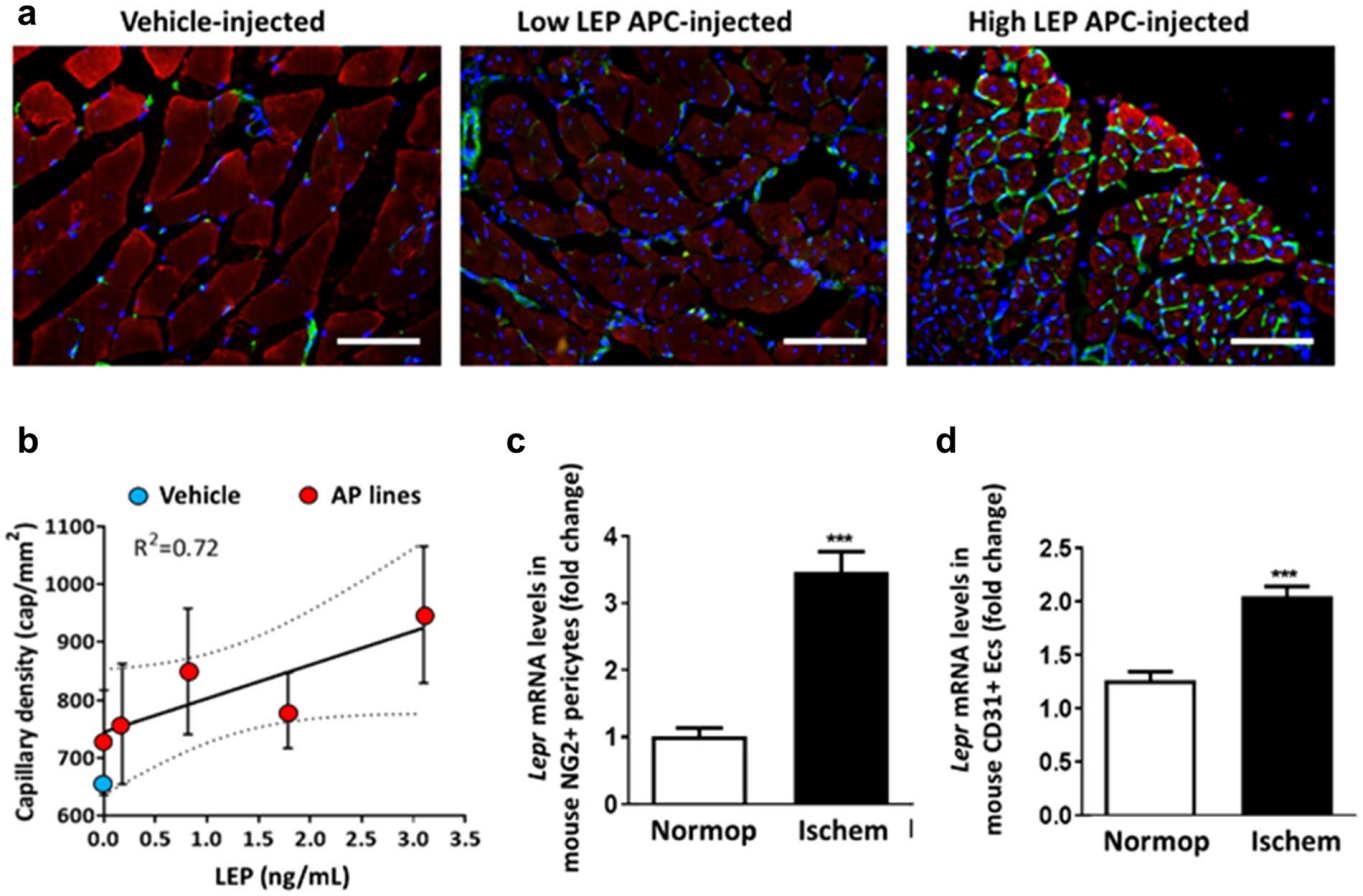
Leptin secretion predicts capillarization outcome of APC therapy in a mouse model of limb ischemia. (**a**) Representative fluorescent microscopy images of capillary density in muscles injected with vehicle or APCs (bar = 50 μm). Capillaries are stained green with isolectin, myocytes stained red with α-SMA, nuclei are stained blue with DAPI. The LEP levels in conditioned media of hypoxic APC lines were assessed by ELISA. APCs producing higher levels of LEP induced more capillarization than those secreting lower levels of LEP. (**b**) Regression line showing the association between capillary density in injected skeletal muscles and level of LEP in conditioned media collected from hypoxic APCs (red circles). Vehicle group capillary density is shown as a light blue circle. (C&D) Vascular pericytes (**c**) and ECs (**d**) immunosorted from mouse adductor muscles 3 days after limb ischemia (Ischem) showed a significant increase in the expression of the mouse *Lepr* compared with contralateral normoperfused (Normop) muscles. ***p < 0.001 *vs*. normoperfused.

APCs transplantation improved the hemodynamic outcome (time-weighted blood flow recovery: 0.48 ± 0.02 Doppler units [min 0.38 ± 0.04, max 0.61 ± 0.08] *vs*. 0.32 ± 0.05 Doppler units in the vehicle-injected group, *p* < 0.01) and also increased capillary density in the ischemic muscle (813 ± 39 cap/mm^2^ [min 727 ± 80, max 947 ± 112] *vs*, 660 ± 12 cap/mm^2^ in the vehicle-injected group, *p* < 0.01). No association was found between the Doppler blood flow data and the capillary density in ischemic muscles. Hence, these parameters were considered as separate outcome indexes in the retrospective analysis of LEP as a predictor of therapeutic efficacy. We did not find any association between LEP levels in the conditioned media of injected APCs and perfusion recovery (R^2^ = 0.35, *p* = N.S.). On the other hand, there was an association between LEP levels in APC conditioned media and capillary density in the ischemic muscles (R^2^ = 0.72, *p* < 0.05, Fig. 6a and b). In contrast, the levels of VEGF-A and Angiopoietin-1 did not correlate with muscle capillarization (data not shown). Thus, the assessment of LEP helps to predict the microvascular outcome of APC therapy in a mouse model of limb ischemia. Furthermore, qPCR analysis of NG2 + pericytes and CD31 + ECs cells extracted from limb muscles of mice 3 days after induction of ischemia showed a significant increase in the expression of the murine *Lepr* in both cell types when compared with cells extracted from contralateral normoperfused muscles (n = 5 per group, *p* < 0.001, Fig. 6c and d).

## Discussion

This study newly shows LEP and its receptor are key players in the functional adaptation of human APCs to a hypoxic environment. Induction of LEP expression by hypoxia inhibits APCs proliferation and apoptosis while augmenting the APCs migratory ability and proangiogenic activity.

Previous reports have shown that hypoxia-preconditioned murine MSCs exert cardio-protective effects in preclinical models through a paracrine mechanism involving the murine *Lep* ^23, 24^. However, the modulation and regulatory roles of LEP in human pericytes has not been investigated so far. Hence, this is the first documentation of LEP being essential for the functional responses of human APCs to hypoxia. LEP is promptly transcribed upon APCs exposure to low oxygen and accumulates in the intracellular compartment, thereby allowing prolonged secretion after cell return to normoxia. The APCs capacity to secrete LEP under hypoxia is exceptional, considering protein levels in the APC-CM are ~10 times higher compared with those reported in the present study and in a previous publications in BM-MSCs^23^. Also, functional analysis of gene arrays indicates that hypoxia triggers a network of proangiogenic and prosurvival genes, which may cooperate with LEP in influencing a spectrum of autocrine and paracrine functions.

The best-characterized molecular mechanism downstream of LEP involves the JAK-STAT3-dependent pathway^27^. Tyrosine-phosphorylated STAT3 activates transcription of target genes, causing negative feedbacks on LEP signalling through the inhibition of LEPR phosphorylation. Likewise, the protein tyrosine phosphatase PTP1B interferes with LEP by dephosphorylating JAK2. However, our study does not show a significant modulation of PTP1B following hypoxia. Additional LEP-dependent pathways include JAK2-induced activation of the insulin receptor substrate/PI3K signalling and protein kinase c-Jun N-terminal kinase. In the heart, the LEP-associated PI3K pathway, along with ERK cascades, plays an important role in protecting cardiac cells from ischemia/reperfusion injury^28, 29^. Moreover, the α and β isoforms of p38 MAPK, which are abundantly expressed in the heart, may mediate the LEP ability to promote cardiomyocyte and vascular smooth muscle cell proliferation and hypertrophy^16^. Also, LEP-induced cardioprotection involves the stimulation of reparative vascularization. *In vitro* and *in vivo* evidence indicates that LEP-induced angiogenesis is dependent on stimulation of VEGF expression^30^ and activation of AKT and ERK^31^ and is synergistic with that of VEGF and FGF^32^. Of note, LEP has been implicated in the mobilization, adhesion to the site of vascular injury and vasculogenic activity of circulating angiogenic cells, *via* NOX2-mediated activation of metalloproteinase-9 and cross-talk of LEPR-SRC Kinase-Integrin αvβ5^33–35^. LEP-induced increase in vascular permeability is essential for cell transmigration across the vasculature and also reportedly favors the release of paracrine factors, including LEP itself, in the circulation^32^. Our study indicates that the canonical STAT3 pathway plays a major role in the functional adaptation of APCs to hypoxia.

We have previously shown that APCs exert a paracrine angiogenic action through VEGF, ANG-1 and miR-132^6^. The present study newly indicates that LEP is another key component of the APC angiogenic secretome. LEP reportedly induces VEGF expression in cancerous cells *via* canonical and non-canonical signalling pathways^36, 37^. However, in the present study, VEGF upregulation by hypoxia was not altered by LEP knockdown (our unpublished data), thus suggesting the prevalence of other stimulatory mechanisms different from the adipokine.

Our research provides important information regarding the putative interaction between LEP and miR-210. A study from Wu’s group showed that forced expression of miR-210 in HL-1 cardiomyocytes by transfection with a lentivirus carrying the miR-210 precursor stimulates the release of angiogenic factors, including Lep^25^. We found that miR-210 silencing does not affect the ability of hypoxic APCs to produce and release LEP. It should be pointed out that, in our study, miR-210 was upregulated by ~14 folds in APCs following induction of hypoxia, whereas, in Wu’s study, pre-miR-210 treatment increased the mature form of miR-210 by 124 fold^25^. A common limitation of the overexpression approach is that it is difficult to glean accurate biological insights when genes are expressed at extremely high, non-physiologic levels. Also, the targets of a specific microRNA may vary among different cells and species.

Clinical and commercial success of cell-based therapies rely on an in-depth understanding of the product’s critical quality attributes, namely the reproducible characteristics that ensure the product has the desired efficacy and safety profile. Therefore, we have established a rigorous production procedure to limit sources of variability associated with the APC manufacturing^1^. Nevertheless, biological variability represents an inevitable feature of cell therapy due to the nature of the products and clinical diversity of donors.

Importantly, re-analysing our previously published study^10^, we found that the abundance of APC-secreted LEP predicts the microvascular benefit, but not the perfusion outcome, of APC transplantation in mice with limb ischemia. We paid attention to avoid common limitations of retrospective studies, including selection bias at enrolment or differential loss to follow up. Nevertheless, our *in vivo* study represents a small scale project, whose aim was to provide initial proof of principle for the *in vitro* findings, thus supporting subsequent larger validation studies prior to guided therapeutic applications of APCs into human subjects. Furthermore, our search for an association between LEP and therapeutic outcomes was not intended to disclose a cause-effect relationship. We have previously shown that numerous paracrine factors contribute to the therapeutic action of human APCs^5, 6, 9^. The finding that ischemia induces the expression of the murine *Lepr* in resident vascular cells suggests favourable environmental conditions to the angiogenic action of LEP-expressing APCs.

## Conclusions

**Figure 7:**
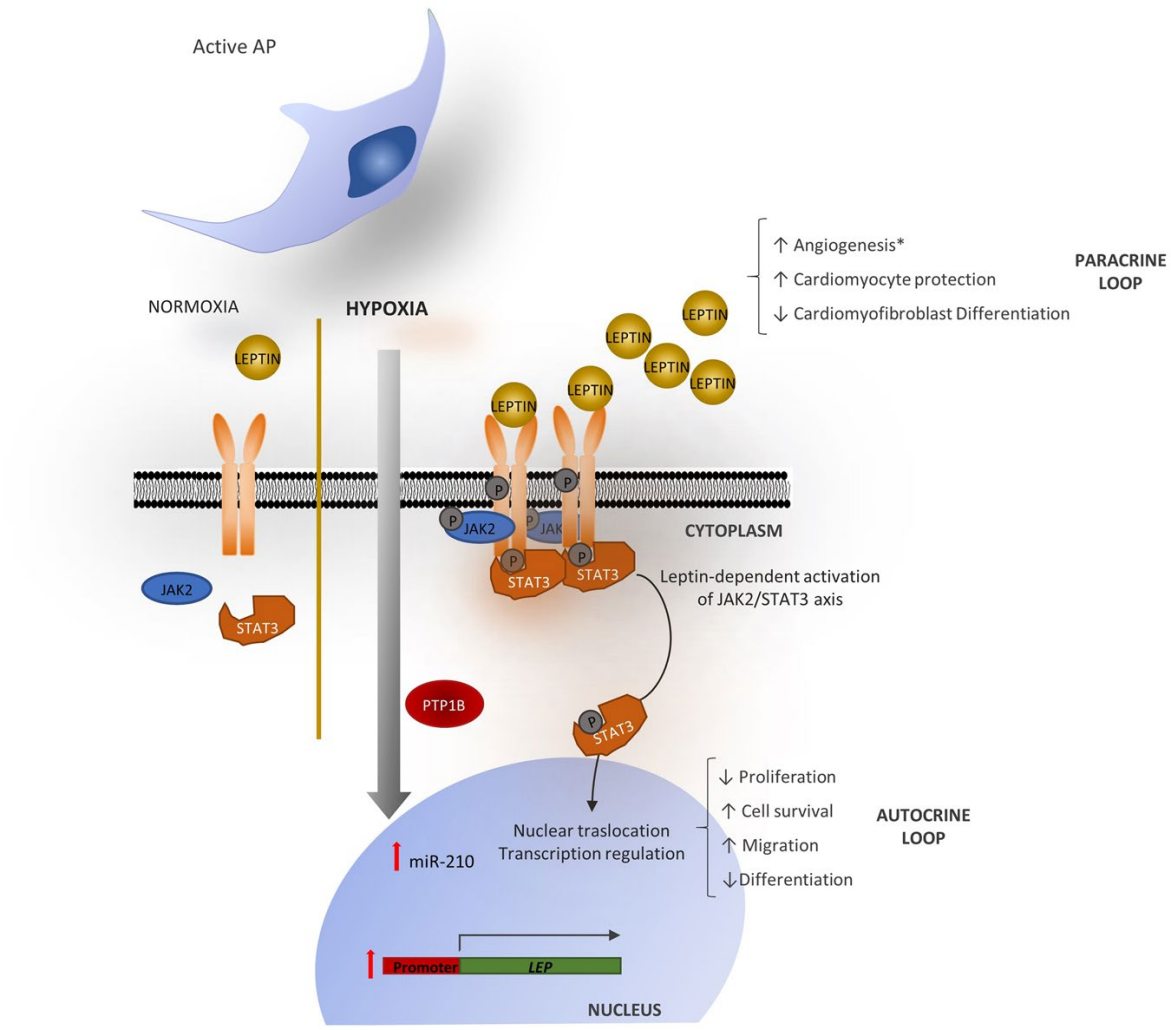
Schematic representation of LEP signalling activated by hypoxia in human adventitial pericytes. An autocrine loop is activated through induced expression and secretion of LEP which binds to the LEPR on pericyte surface. The paracrine loop is mediated by secreted LEP inducing functional responses in other cells, like endothelial cells. The autocrine and paracrine loops result in functional adaptations to hypoxia. Arrows indicate enhanced or reduced function. Hypoxia activates miR-210, which in other cell types activates LEP production and PTP1B-mediated dephosphorylation of pJAK2. However, in human APCs, miR-210 is not responsible for modification of LEP secretion or signalling.

This is the first study to report the functional importance of the LEP signalling pathway in human pericytes from the vascular adventitia. LEP is strongly upregulated by hypoxia, and this transcriptional modification confers pericytes with resistance to apoptosis and enhanced migratory and angiogenic activity (Fig. 7). These results have important implications for therapeutic revascularization of ischemic tissues and may also provide far-reaching interpretation of LEP-regulated processes involving perivascular cells, including vascular remodelling, atherosclerosis, and cancer.

## Material and Methods

### Ethics

Studies on cells from human subjects complied with the principles stated in the “Declaration of Helsinki” and were covered by ethical approvals (06/Q2001/197 and 14/WA/1005 for APC and BM-MSC studies, respectively, released by the National Institute for Social Care and Health Research – Research Ethics Service). All the subjects gave informed written consent for the experimental use of donated material. APCs were isolated from vein leftovers of coronary artery bypass graft surgery patients, whose characteristics are reported in Supplementary Table I. In addition, BM-MSCs were isolated from bone surgical leftovers of patients (n = 3, age 35–65 years), undergoing orthopaedic surgery for hip replacement at the Avon Orthopaedic Centre (Southmed Hospital, UK). Mononuclear fractions collected after Ficoll centrifugation were immuno-sorted using CD34/CD45 microbeads (Miltenyi Biotec). BM-MSCs were used as control for LEP release since they are the current ‘gold standard’ for cell therapy and regenerative medicine.

Experiments involving live animals were performed in accordance with the Guide for the Care and Use of Laboratory Animals (the Institute of Laboratory Animal Resources, 1996) and with the approval from the British Home Office and the University of Bristol. Analysis of historical results followed the guidelines for the best practices for use of experimental data in laboratory animals^38, 39^.

### Standard operating protocol for isolation and culture of human APCs

Cells were isolated and expanded from vein leftovers to obtain a total of 21 APC lines, using a standard operating protocol described in previous publications^5, 10^. Purity of the cell preparations was assessed by flow cytometry and immunocytochemistry. Experiments were performed at passage 7 (P7). We followed a randomization schedule for attribution of individual cell lines to different assays to eliminate the selection bias and avoid confounding from known and unknown factors.

### Induction of hypoxia and collection of conditioned media

Confluent APCs were exposed to normoxia (20% O2) or hypoxia (2% O2) for the time durations described in the result sections. FBS-free EGM-2 media was used for the majority of studies, and it was also depleted of VEGF (VEGF/FBS-free EGM-2) in trans-endothelial electrical resistance assays. Morphological features of APCs were captured using a Nikon eclipse TS100 microscope, at 4x and 10x magnification. Cellular RNA and protein were collected using standard methods described below. CM were centrifuged at 12,000 *g* for 5 min to eliminate debris and floating cells and stored at −80 °C until assayed. Additionally, BM-MSCs were exposed to normoxia and hypoxia (20% and 2% O_2_, respectively) for 48 h. FBS-free MEM media was used for the studies in BM-MSCs including collection of CM and RNA which were used thereafter in ELISA and qPCR studies

### Flow cytometry analyses

APCs were stained for surface antigen expression using combinations of the following antibodies to confirm typical phenotype: CD105 (Life Technologies), CD90, CD73, PDGFRβ (Biolegend), CD44 (eBioscience), CD31 (BD Biosciences), CD34, and CD45 (Miltenyi)^5, 10^. Analysis was performed using a FACS Canto II flow cytometer and FACS Diva software (both BD Biosciences). BM-MSCs antigenic characterization was also routinely performed by flow cytometry. Primers and antibodies are listed in Supplementary Tables VI and VII, respectively.

### Immunocytochemistry

Cells were fixed with freshly prepared 4% PFA and probed with the following antibodies – NG2 (1:100, Millipore), PDGFRβ (1:50, Santa Cruz), GATA4 (1:100), (all Abcam) followed by Alexafluor 488 anti-rabbit or anti-mouse secondary antibodies (Invitrogen). Always, isotype negative controls were performed to ensure the immunofluorescence specificity. Cell nuclei were stained with 300 nM 4′,6-diamidino-2-phenylindole dilactate (DAPI) (ThermoFisher Scientific). Images were acquired with a fluorescent microscope (Olympus BX40) at 40x magnification and merged using ImageJ software.

### Gene arrays and bio-function analysis

Labelled cRNA was hybridised onto 4 × 44 k Agilent whole human genome microarrays overnight, washed and scanned, and the obtained 16 bit tiff images were analysed using Agilent Feature Extraction software. All steps were performed according to manufacturer’s instructions (Agilent Technologies).

### Assessment of mRNA or microRNA expression by PCR

Total RNA was isolated using Trizol Reagent (Life Technologies) and miRNeasy mini kit (Qiagen) following the manufacturer’s instructions. RNA concentration and purity was assessed using a NanoDrop 2000 Spectrophotometer (ThermoFisher Scientific). Total RNA was reverse transcribed using a high-capacity RNA-to-cDNA kit for mRNA analysis, or using specific Taqman microRNA assay primers with a TaqMan® MicroRNA Reverse Transcription Kit for the assessment of microRNA expression (both Life Technologies). QPCR was performed using a Light Cycler 480 (Roche). Targeted genes are listed in Supplementary Table VI. Data were analysed using the 2^−ΔΔCt^ method. The relative expression of each selected gene product was calculated by comparing the negative log of the Ct of the sample minus the Ct of the control gene (18 s or UBC) or microRNA (U6) in the same sample.

### Gene silencing

Transient inhibition of the *LEP* or *LEPR* gene expression was performed using small interfering RNA (siRNA) molecules (ON-TARGETplus SMARTpool kit, GE Dharmacon). Control cells were exposed to scramble siRNA sequences (Silencer® Select Negative Control No. 2 siRNA, ThermoFisher Scientific). To investigate the relationship between microRNA-210 and the LEP signalling pathway, we transfected human APCs with hsa-miR-210-3p anti-miR (AM10516). Reference APCs received anti-miR negative control (AM17010, both Life Tech). Transfection complexes were prepared using Opti-MEM media (ThermoFisher Scientific), Lipofectamine 2000 (Invitrogen) and siRNA, anti-miR or control sequences following the manufacturer’s instructions. After 6 h, the lipocomplexes were removed, and fresh FBS or FBS/VEGF EGM2 was added as described above. Silencing efficiency was confirmed by qPCR and Western blot methods (see below).

### Protein extraction

Cells were lysed in RIPA buffer containing phosphatase (1:50) and proteinase inhibitors (1:100, both Sigma). After incubation on ice, the whole APC lysates were centrifuged at 12,000 *g* for 10 min at 4 °C. Protein concentration was quantified using a BCA protein assay (ThermoFisher Scientific).

### ELISA

LEP and VEGF levels in conditioned media and cellular lysates were quantified using Human Lep Duo Set ELISA (R&D Systems) following the manufacturer’s instructions. Protein lysates from APC lines from miR210 inhibition experiments were tested by ELISA assay to investigate the regulation of pJAK2 Tyr 1007/1008 (Ray Biotech, Cambridge Bioscience).

### Western blotting

Protein samples were prepared in Laemmli loading buffer, incubated for 5–10 min at 95 °C, resolved on 7.5% SDS-PAGE and transferred onto PVDF membranes (Immobilon-P PVDF Transfer Membrane 0.45 m, Millipore). Membranes were blocked using 5% BSA or 5% non-fat dried milk in TBS 0.05% Tween 20 (Sigma-Aldrich) for 1 h at 15–25 °C. Primary antibodies included pAKT Ser473, total AKT, pERK 1/2 Thr 202/204, total ERK, PTP1B, total STAT3 (clone 124H6) (all 1:1000, Cell Signalling), and pSTAT3 Tyr705 (clone D3A7, 1:2000, Cell Signalling), while -actin (1:5000, Clone AC15, Sigma-Aldrich) was used as loading control. Given the different sizes for AKT (60 KDa), STAT3 (80 KDa), ERK 1/2 (42–44 KDa), PTP1B (50 KDa) and actin (42 KDa), phosphorylated proteins were firstly assessed and the total protein was assessed thereafter. No more than two stripping procedures were performed in the same membrane (RestoreTM Plus Western Blot Stripping Buffer, Thermo Scientific). Anti-rabbit IgG or anti-mouse IgG were employed as secondary antibodies (both 1:5000, GE Healthcare, Fisher Scientific). Membrane development was performed by an enhanced chemiluminescence-based detection method (ECL™ Prime Western Blotting Detection Reagent, GE Healthcare, Fisher Scientific) usually in a ChemiDoc MP system (Bio-Rad). Blot densitometry was analysed by using the Image J 5.1 software. In order to improve the clarity and conciseness of the blot data, they are displayed cropped in the text figures while original images are shown in Supplementary Material (Supplementary Figure V).

### *In vitro* functional assays

Studies were conducted on human APCs exposed to exogenous recombinant human LEP (#398-LP-01M, R&D Systems) or inhibitors of the LEP system. Cell proliferation and apoptosis were measured using BrdU immunofluorescence (Roche) and CaspaseGlo 3/7 (Promega) assays, respectively, according to the manufacturer’s instructions. Cell migration was assessed by the scratch assay as described previously^10^. The Matrigel network formation by HUVECs was performed as described^10^. Network formation was stimulated with the conditioned media of human APCs preliminarily exposed to normoxia or hypoxia. Images were taken under bright-field at 5x, and the number of branches, length, and thickness of the networks was measured using the ImageJ software. Real-time measurement of trans-endothelial electrical resistance by an automated electrical cell impedance sensing system (ECIS 1600R, Applied Biophysics) was employed to determine the effect of human APCs conditioned media on endothelial permeability. Briefly, HUVECs were seeded into 8-well arrays (Ibidi), at a density of 100,000 cells/well, and incubated for 24 h in EGM2 media. After 30 min stabilization, the HUVEC media was replaced with either conditioned media from human APCs preliminarily transfected with leptin silencing sequences or scramble control sequences. Alternatively, the LEP signalling was blocked in HUVECs by *LEPR* siRNA silencing and the above media added. Resistance was measured every 15 sec for 2 h.

### *In vivo* experiments

We performed a retrospective analysis of LEP data from our recent published study, investigating the effect of five human APC lines in C57BL/6 mice with unilateral limb ischemia^10^. Unilateral limb ischemia was performed by ligation and electrical cauterization of the left femoral artery. This was followed by injection of human APCs (8 × 10^4^ cells in 30 μL, P7) or PBS (vehicle) into 3 different points of the operated adductor muscle (n = 7 animals *per* group). In this re-assessment of the study, we determined if the levels of LEP in CM of human APCs allow predicting the cell capacity to stimulate angiogenesis in ischemic muscles. Details of the methodology to assess capillary angiogenesis is provided in ref. 10.

### Isolation of endothelial cells and pericytes from mouse ischemic limb muscles

Adductor muscles from mice at 3 days post-ischemia induction were rinsed and digested with collagenase II (Worthington) plus DNase I (Sigma) using gentleMACS™ Dissociator, following the manufacture’s protocol. Next, pericytes and ECs were immunomagnetically sorted using NG2 and CD31 antibodies, respectively (Miltenyi Biotech). Purity of cell preparations was analyzed by flow cytometry.

### Statistical Analysis

Analyses and graph drawings were performed using the SPSS 19.0 for Windows (SPSS, Inc., Chicago, IL, USA) and GraphPrism 6.0 statistical packages. Continuous variables were tested for normal distribution by Kolmogorov–Smirnov Z test and shown as mean ± standard error of the mean (SEM) or median (IQR). Categorical variables and flow cytometry events are presented as percentages. Continuous variables with normal distribution were compared using the t-Student (two group comparison) or one-way analysis of variance (ANOVA for multiple group comparisons), as appropriate. In specific studies, two-way ANOVA was applied to compare the mean differences between groups that have been split into two independent variables and verify if there was an interaction between the two independent variables on the dependent variable. Homoscedasticity was assessed with the Levene test, and posthoc analysis of ANOVA included Tuckey or Bonferroni and T3 Dunnet, as appropriate. Non-parametric tests including the Mann–Whitney U test or the Kruskall-Wallis test were used for samples not normally distributed. Correlation between variables and microvascular endpoints was calculated by regression analysis. Processing and statistical analysis of gene arrays were done in R/Bioconductor5 and gene set enrichment was performed using ClueGO 6 and GATHER. Statistical significance was accepted at *p* < 0.05.

### Electronic supplementary material

supplementary information
