# Author Correction: The adipokine leptin modulates adventitial pericyte functions by autocrine and paracrine signalling

**DOI:** 10.1038/s41598-024-56932-3

**Published:** 2024-03-26

**Authors:** Federica Riu, Sadie C. Slater, Eva Jover Garcia, Iker Rodriguez-Arabaolaza, Valeria Alvino, Elisa Avolio, Giuseppe Mangialardi, Andrea Cordaro, Simon Satchell, Carlo Zebele, Andrea Caporali, Gianni Angelini, Paolo Madeddu

**Affiliations:** 1 Bristol Heart Institute, School of Clinical Sciences, University of Bristol, Bristol Royal Infirmary, Bristol, BS2 8HW UK; 2 Division of Cancer and Stem Cells, School of Medicine, Cancer Biology, University of Nottingham, Nottingham, NG7 2UH UK; 3 Centre for Cardiovascular Science, Queen’s, Medical Research Institute, Edinburgh, EH16 4TJ UK

Correction to: *Scientific Reports* 10.1038/s41598-017-05868-y, published online 14 July 2017

The original Article contains an error in Figure 5b assembly where the figure in panel “HUVEC + CM” was a duplication of a figure in panel “HUVEC CONTROL”.

The corrected Figure 5 and accompanying legend appear below as Figure 5.

**Figure 5:**
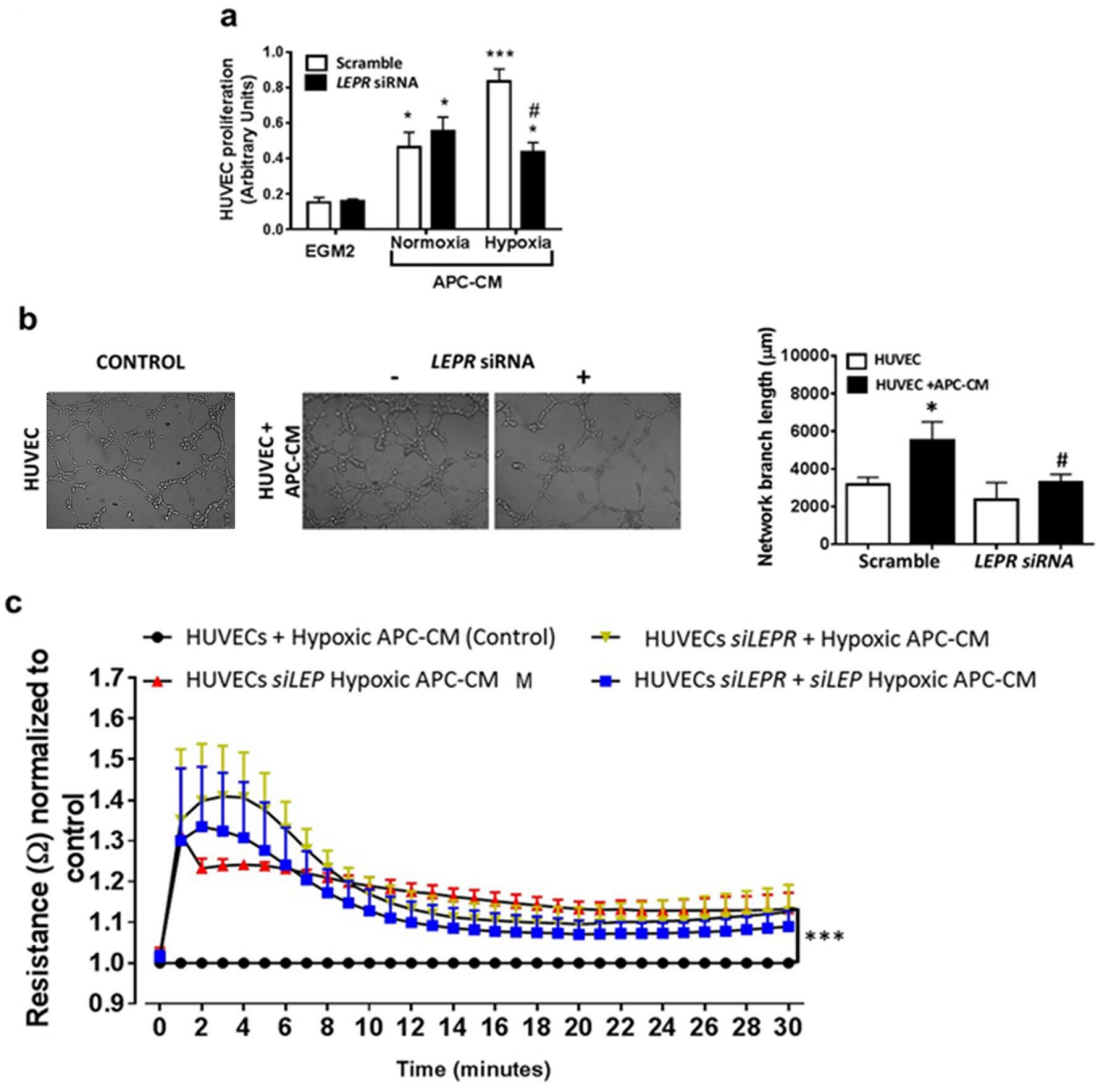
Angiocrine activity of APC-secreted LEP (**a**) *LEPR* silencing inhibited the proliferative effect of hypoxic APC-CM on HUVECs. *p < 0.05 and ***p < 0.001 versus EGM2 ^#^*p* < 0.05 versus scramble. (**b**) *LEPR* silencing abolished the promotion of HUVEC network formation by hypoxic APC-CM. *p < 0.05 versus EGM2 ^#^*p* < 0.05 versus scramble. (**c**) Blockade of *LEP* signalling increases the vascular resistance of HUVEC monolayers exposed to hypoxic APC-CM. ***p < 0.001 versus control.

