# Supplementary material for: The adipokine leptin modulates adventitial pericyte functions by autocrine and paracrine signalling
Source: Sci Rep. 2017 Jul 14;7:5443. doi: 10.1038/s41598-017-05868-y (PMC5511138; doi:10.1038/s41598-017-05868-y)
Supplement: Supplementary file 1 — supplementary information [file 41598_2017_5868_MOESM1_ESM.pdf]

Supplementary material

The adipokine leptin modulates adventitial pericyte functions by autocrine and paracrine signalling

First author: Riu

Running title: Pericytes signal through the adipokine leptin

***Federica Riu PhD[1,2], *Sadie C. Slater PhD[1], *Eva Jover Garcia PhD[1], Iker Rodriguez-Arabaolaza MSc[1], Valeria Alvino MSc[1], Elisa Avolio PhD[1], Giuseppe Mangialardi MD PhD[1], Andrea Cordaro[1], Simon Satchell PhD[1], Carlo Zebele MD[1], Andrea Caporali PhD[2], Gianni Angelini MD[1], and Paolo Madeddu MD[1].**

Supplementary Table I. Clinical and demographic characteristics of the 21 donors from which we have successfully isolated and expanded APCs for this study.

**Baseline characteristics**	
Male sex, n (%)	21 (100)
Age, median [IQR]	64 [58-71]
Hypertension, n	18
Diabetes mellitus, n	1
Hyperlipidaemia, n	17
Body mass index (kg/m2), median [IQR]	32 [26-34]
Smoking habit, n	
Previous	3
Current	13
Previous Myocardial Infarction	13 (54.2)
Coronary Artery Disease, n	
1 vessel affected	1
2 vessels affected	7
3 vessels affected	13
Angina classification (CSS score), n	
I	3
II	4
III	9
IV	2
NYHA classification, n	
I	5
II	9
III	1

CSS (Canadian Cardiovascular Society) Functional Score

Supplementary Table II. Expression of surface markers in human APCs as assessed by flow cytometry

Condition	APC line	CD90	CD73	CD140b	CD44	CD34	CD105	CD31	CD45
20% O2	7.05.14 A	88	93	40	98	0.50	40	0.10	1.50
	28.10.14 C	86	95	42	99	0.10	77	0.10	1.30
	8.05.14 D	80	82	43	98	0.70	86	0.80	1.50
	13.10.15 E	68	98	47	97	0.60	93	0.80	0.20
**Mean**		**81**	**92**	**43**	**98**	**0.48**	**74**	**0.45**	**1.13**
**SEM**		**5**	**4**	**1**	**1**	**0.13**	**12**	**0.20**	**0.31**

Condition	APC line	CD90	CD73	CD140b	CD44	CD34	CD105	CD31	CD45
2% O2	7.05.14 A	91	90	33	99	0.60	43	0.10	0.90
	28.10.14 C	89	93	40	99	0.10	75	0.10	0.60
	8.05.14 D	70	89	50	98	0.70	84	0.70	1.50
	13.10.15 E	72	98	51	97	0.70	81	1.10	3.20
**Mean**		**81**	**93**	**44**	**98**	**0.53**	**71**	**0.50**	**1.55**
**SEM**		**6**	**2**	**4**	**1**	**0.14**	**9**	**0.24**	**0.58**

Supplementary Table III. List of the top genes modulated by hypoxia in human APCs

	GENENAME	GOCC	logFC	P.Value
1.	*SPAG4*	spermatogenesis	3.705932	0.002295
2.	*PDK1*	carbohydrate metabolic process	2.285972	3.74E-09
3.	*AHNAK*	nervous system development	2.067459	4.06E-07
4.	*SLC2A1*	carbohydrate metabolic process	2.044775	2.80E-07
5.	*CRYM*	negative regulation of transcription	1.99988	0.000448
6.	*ABCD3*	peroxisome organization	1.894208	2.00E-06
7.	*TERF1*	G2/M transition of mitotic cell cycle	1.851164	0.00339
8.	*CCT6B*	protein folding	1.838416	0.006236
9.	*LEP*	metabolism/angiogenesis	1.819224	0.006227
10.	*OSCP1*	transport	1.730382	0.003012
11.	*LHX3*	regulation of transcription, DNA-dependent	1.728171	0.003373
12.	*ETNK1*	phosphatidylethanolamine biosynthetic process	1.706657	1.20E-05
13.	*CTDP1*	transcription	1.614284	0.008019
14.	*ENO2*	carbohydrate metabolic process	1.572633	1.52E-06
15.	*OR13F1*	sensory perception of smell	1.567926	0.003639
16.	*GYS1*	carbohydrate metabolic process	1.534586	1.46E-05
17.	*PFKFB4*	carbohydrate metabolic process	1.498669	0.007964
18.	*ALDOC*	response to hypoxia / apoptosis	1.489168	0.001565
19.	*ARHGEF1*	Rho protein signal transduction	1.452935	0.001115
20.	*B3GALT4*	protein glycosylation	1.425453	0.009055
21.	*ADSSL1*	immune system process	1.414419	0.000668
22.	*CCL28*	chemotaxis / immune response	1.3827	0.002895
23.	*MED23*	regulation of transcription, DNA-dependent	1.38237	3.19E-05
24.	*TRIOBP*	barbed-end actin filament capping modification	1.382094	5.81E-05
25.	*NXPH4*	neuropeptide signaling pathway	1.37048	0.000312
26.	*CIRBP*	response to cold / response to UV	1.34684	5.00E-05
27.	*PTPRR*	in utero embryonic development tion	1.342777	0.002684
28.	*RASGRP2*	regulation of cell growth / blood coagulation	1.326673	0.001154
29.	*BNIP3*	response to hypoxia / apoptosis	1.325714	4.96E-06
30.	*TREM1*	blood coagulation / intracellular signal transduction	1.303737	0.000893

31.	*TCEA2*	transcription elongation, DNA-dependent	1.287599	0.001964
32.	*SNTA1*	muscle contraction	1.28359	0.000218
33.	*MPP2*	signal transduction	1.244355	0.00699
34.	*CMTM1*	chemotaxis	1.240975	0.000109
35.	*GCHFR*	protein complex assembly	1.21647	0.009382
36.	*LDHA*	pyruvate metabolic process / glycolysis	1.212298	6.16E-05
37.	*UBE3B*	protein ubiquitination involved	1.206514	0.001477
38.	*SLC6A8*	muscle contraction / creatine metabolic process	1.204214	0.000358
39.	*HMHA1*	small GTPase mediated signal transduction	1.192093	0.002556
40.	*CTNNBIP1*	regulation of vascular permeability	1.182575	0.000431
41.	*PFKL*	carbohydrate metabolic process	1.167166	0.000667
42.	*PKP3*	cell adhesion	1.161209	0.000116
43.	*FBXO2*	protein ubiquitination	1.15519	0.006687
44.	*CARHSP1*	intracellular signal transduction	1.152805	0.004374
45.	*SLC22A18AS*	biological_process	1.145768	0.000668
46.	*SLC16A3*	blood coagulation / pyruvate metabolic process	1.145418	0.002141
47.	*AANAT*	metabolic process	1.130998	0.000191
48.	*NAT6*	metabolic process	1.12475	0.002932
49.	*TPI1*	carbohydrate metabolic process	1.109328	0.002914
50.	*MAST1*	cytoskeleton organization / protein phosphorylation	1.105299	0.004676
51.	*GDPD5*	glycerol metabolic process / lipid metabolic process	1.101075	0.00466
52.	*ADRA2C*	blood coagulation / energy reserve metabolic process	1.100074	0.004386
53.	*WIPF1*	actin cytoskeleton organization	1.089793	0.000387
54.	*ALDH3B1*	alcohol metabolic process	1.088886	0.006517
55.	*ACOT1*	very long-chain fatty acid metabolic process	1.076034	0.00055
56.	*BEST4*	ion transport / biological_process	1.065031	0.001634
57.	*DACH2*	multicellular organismal development	1.064606	0.009908
58.	*ZNF680*	regulation of transcription, DNA-dependent	1.058634	0.006961
59.	*RPP30*	tRNA processing	1.058132	0.000732
60.	*SPINT2*	cellular component movement	1.058098	0.004402
61.	*DYNC2LI1*	multicellular organismal development	1.036759	0.002102
62.	*VEGFA*	angiogenesis / vasculogenesis	1.033029	0.002273

63.	*KIAA1456*	metabolic process	1.030766	0.003575
64.	*GABRA3*	gamma-aminobutyric acid signaling pathway	1.021465	0.002434
65.	*SSH2*	actin cytoskeleton organization	1.020271	0.000156
66.	*TLE1*	signal transduction	1.018402	0.000759
67.	*PKN1*	signal transduction / protein phosphorylation	1.015443	0.000155
68.	*TCF25*	negative regulation of transcription	1.012701	0.000851
69.	*CCDC85B*	negative regulation of transcription	1.009642	0.002349
70.	*CLCN4*	transport / ion transport	1.004777	0.00134
71.	*MAP1LC3A*	autophagic vacuole assembly / autophagy	1.003569	0.009447
72.	*RASSF7*	signal transduction	1.002955	0.003799
73.	*ACSL3*	fatty acid biosynthetic process	-1.00193	0.0018
74.	*SERPINH1*	response to unfolded protein	-1.00277	0.000442
75.	*STRBP*	cellular component movement	-1.00416	0.004286
76.	*MANF*	response to unfolded protein biological_process	-1.0043	0.000272
77.	*ZNF605*	regulation of transcription, DNA-dependent	-1.00537	0.001741
78.	*PMS1*	ATP catabolic process	-1.0111	0.007456
79.	*CHORDC1*	chaperone-mediated protein folding	-1.01377	0.009586
80.	*ZNF727*	regulation of transcription, DNA-dependent	-1.01591	0.003506
81.	*CTSL1*	proteolysis	-1.02335	0.000623
82.	*HIF1A*	response to hypoxia	-1.02371	0.006344
83.	*DEPDC1*	intracellular signal transduction	-1.02523	0.009741
84.	*CLK4*	peptidyl-tyrosine phosphorylation	-1.0358	0.006531
85.	*HNRNPA2B1*	nuclear mRNA splicing, via spliceosome	-1.04691	0.003321
86.	*ZNF37A*	transcription, DNA-dependent	-1.04826	0.004272
87.	*KHDRBS3*	spermatogenesis / regulation of transcription	-1.05998	0.005251
88.	*NEK10*	protein phosphorylation	-1.06045	0.003277
89.	*HECTD2*	protein ubiquitination	-1.06068	0.003356
90.	*ZNF280D*	regulation of transcription	-1.06133	0.007403
91.	*LUC7L*	negative regulation of muscle tissue development	-1.06208	0.000816
92.	*TMX3*	cell redox homeostasis	-1.06818	0.009781
93.	*TBCK*	regulation of Rab GTPase activity	-1.06972	0.000216
94.	*ABCC5*	transport	-1.09073	0.000218

95.	*TRNT1*	RNA processing	-1.09174	0.001091
96.	*TLR3*	toll-like receptor signaling pathway	-1.09883	0.005746
97.	*NAMPT*	signal transduction	-1.09971	0.005848
98.	*DBT*	metabolic process	-1.10102	0.000119
99.	*NUDT3*	cell-cell signaling	-1.10319	0.000218
100	*CCDC76*	tRNA processing	-1.11069	0.00552
101	*TBC1D8B*	regulation of Rab GTPase activity	-1.11681	0.006732
102	*TET3*	oxidation-reduction process	-1.11892	0.002769
103	*HIBCH*	branched chain family amino acid catabolic process	-1.1232	0.000546
104	*C5*	activation of MAPK activity g pathway	-1.12359	0.000257
105	*PRPF4B*	nuclear mRNA splicing, via spliceosome	-1.13006	0.004421
106	*HMGB3*	DNA recombination	-1.13404	0.001608
107	*CASP1*	apoptosis	-1.1451	0.003074
108	*ARHGAP5*	cell adhesion	-1.14673	0.006035
109	*RIC8B*	regulation of G-protein coupled receptor protein	-1.14797	0.002709
110	*TIA1*	apoptosis	-1.14852	0.001733
111	*B3GNT6*	protein glycosylation	-1.16122	0.008494
112	*LYG1*	metabolic process / peptidoglycan catabolic process	-1.18663	0.001265
113	*LUC7L3*	apoptosis / mRNA processing	-1.21856	0.003643
114	*ZRANB2*	transcription, DNA-dependent / mRNA processing	-1.22432	0.003998
115	*TAF9B*	transcription initiation, DNA-dependent	-1.23045	0.000572
116	*FMN1*	actin cytoskeleton organization	-1.24468	0.008358
117	*ZNF148*	negative regulation of transcription	-1.24485	0.000852
118	*L3MBTL1*	regulation of cell cycle	-1.24825	0.003229
119	*HERC4*	protein ubiquitination	-1.27386	0.002253
120	*N4BP2L2*	cell killing / biological_process	-1.30173	0.001487
121	*TFRC*	response to hypoxia	-1.33116	0.003532
122	*CCDC41*	biological_process	-1.34177	0.003825
123	*SSR1*	cotranslational protein targeting to membrane	-1.38737	0.00834
124	*OMA1*	proteolysis	-1.40119	0.000229
125	*IFNAR1*	JAK-STAT cascade	-1.4955	0.001177
126	*HS3ST3B1*	heparan sulfate proteoglycan biosynthetic process	-1.51249	0.008323

127	*MTERFD3*	regulation of transcription	-1.64193	0.008759
128	*PREPL*	proteolysis	-1.66411	0.001247
129	*EIF4A2*	nuclear-transcribed mRNA catabolic process	-1.76099	9.18E-05
130	*HIST1H2AC*	nucleosome assembly	-1.8072	0.006223
131	*GLIS3*	regulation of transcription	-1.9221	0.004129

Supplementary Table IV. List of the top functions associated with networks modulated by hypoxia in human APCs.

ID	Score	Focus Genes	Top Functions
1	50	28	Cell Cycle
2	47	28	Carbohydrate Metabolism
3	29	19	Cell Death/apoptosis and cell-to-cell signalling/interaction

LEP is the central core of Network 3**. Score** = Total genes in network; **Focus Genes** = Genes regulated in dataset. Details of the 19 genes modulated network 3 are given in Supplementary Table V.

Supplementary Table V. List of genes comprised in the network 3 identified by IPA

Symbol	Full Name	Aliases
AANT	Aralkylamine N Acetyltransferase	DSPS, SNAT, AANAT
ACSL3	AcylCoA Synthetase Long Chain Family Member 3	ACS3, PRO2194, FACL3, LACS3
CASP1	Caspase 1, Apoptosis Related Cysteine Peptidase	p45, caspase1, beta, convertase, IL1B convertase, IL1BC, ICE, CASP1, IL1BC, IL1BCE
CTSL1	Cathepsin L1	EC 3.4.22.15, MEP, CATL
CXCL2	Chemokine (CXC Motif) Ligand 2	MIP2A, MIP2A, MIP2, CINC2a, GROb, MGSAb, grobeta, GRO2, MIP2alpha, SCYB2
HS3ST3B1	Heparan Sulfate (Glucosamine) 3O Sulfotransferase 3B1	3OST3B, 3OST3B1,h3OST3B, 30ST3B1, HS3ST3B
IRGM	Immunity Related GTPase Family, M	IRGM1, IFI1, LRG47, LRG47, IBD19
IVNS1ABP	Influenza Virus NS1A Binding Protein	ND1, NS1, HSPC068, KLHL39, NS1, NS1BP, FLARA3,
KIF20A	Kinesin Family Member 20A	rabkinesin6, RAB6KIFL, GG10_2, MKLP2
LEP	Leptin	OB, leptin, OBS, LEPD
MARK4	MAP/Microtubule Affinity Regulating Kinase 4	MARK4L, MARK4S, MARKL1L,
NME7	NME/NM23 Family Member 7	CFAP67, NDK7, MN23H7, nm23H7
PLA2G4C	Phospholipase A2, Group IVC (Cytosolic, Calcium Independent)	CPLA2gamma
SERPINH1	Serpin Peptidase Inhibitor, Clade H (Heat Shock Protein 47), Member 1, (Collagen Binding Protein1)	PIG14, RAA47, colligin1, colligin2, gp46, OI10, PPROM, CBP1, CBP2, SERPINH2, AsTP3, HSP47, Colligin
TREM1	Triggering Receptor Expressed On Myeloid Cells 1	CD354, TREM1
VEGFA	Vascular Endothelial Growth Factor A	MVCD1, VEGF, VPF, VEGFA
WIPF1	WAS/WASL Interacting Protein Family, Member 1	PRPL2, WAS2, WASPIP, WIP
ZEB1	Zinc Finger EBox Binding Homeobox 1	BZP, DELTAEF1, ZFHEP, ZFHX1A, FECD6, NIL2A, PPCD3, TCF8, AREB6, TCF8
ZRANB2	Zinc Finger, RAN Binding Domain Containing 2	ZIS1, ZIS2, ZNF265, ZIS

Upregulated (**red**) and downregulated genes (**green**)

Supplementary Table VI. Primers and probes used to assess gene expression by qPCR

Target gene	Commercial information	qPCR assay	Nomenclature/ Function
LEP	QT000030261 (QIAGEN); Hs00174877 (Applied Biosystems)	SYBR Green; Taqman	Leptin
LEPR	QT00006524 (QIAGEN); Hs00174497 (Applied Biosystems)	SYBR Green ; Taqman	Leptin receptor
18S	QT001969367 (QIAGEN)	SYBR Green	Housekeeping genes
UBC	Hs1871556 (Applied Biosystems)	Taqman	
MIR210	00512 (Applied Biosystems)	Taqman	hsa-miR-210-3p
snRNAU6	001973 (Applied Biosystems)	Taqman	miRNA housekeeping

Supplementary Table VII. Catalogue numbers of antibodies used

Target protein	Assay	Catalogue number	Supplier
PDGFRβ	IF	sc-339	Santa Cruz
NG2	IF	AB5320	Millipore
GATA-4	IF	ab61767	Abcam
Isolectin B4	IF	I21414	Invitrogen
α-SMA	IF	C6198	Sigma-Aldrich
Alexafluor488 Anti-Rabbit	IF	A-11008	Invitrogen
Alexafluor488 Anti-mouse	IF	A-11001	Invitrogen
p-STAT3 (tyr705)	Western blot	9145 (D3A7)	Cell Signalling
Total STAT3	Western blot	9139 (124H6)	Cell Signalling
p-AKT	Western blot	9271	Cell Signalling
Total AKT	Western blot	9272	Cell Signalling
p-ERK1/2 (Thr 202/204)	Western blot	9101	Cell Signalling
Total ERK1/2	Western blot	4695 (137F5)	Cell Signalling
PTP1B	Western blot	5311	Cell Signalling
β-actin	Western blot	A5441 (AC15)	Sigma Aldrich
Leptin receptor	Western blot	ab104403	abcam
Anti-Rabbit IgG	Western blot	NA934	GE Healthcare
Anti-mouse IgG	Western blot	NA931	Ge healthcare
CD105	FACS	MHCD10505	Life technologies
CD90	FACS	328124	Biolegend
CD73	FACS	344010	Biolegend
PDGFRβ	FACS	323606	Biolegend
CD44	FACS	17-0441-82	eBiosciences
CD31	FACS	555445	BD Biosciences
CD34	FACS	130-081-001	Miltenyi
CD45	FACS	130-094-975	Miltenyi
NG2	FACS	8012-6504-120	eBioscience
NG2	Cell sorting	130-097-171	Miltenyi

Supplementary Figure I: APCs morphology (a, contrast phase microscopy) and immunocytochemistry images of typical APC antigens captured 40x (b). Typical histograms of flow cytometry data from a representative cell line under normoxia (c) and hypoxia (d). Average values of markers expression (e). Individual and average data of four cell lines are also shown in Supplementary Table II. Expression of these typical markers is similar in APCs exposed to normoxia or hypoxia.

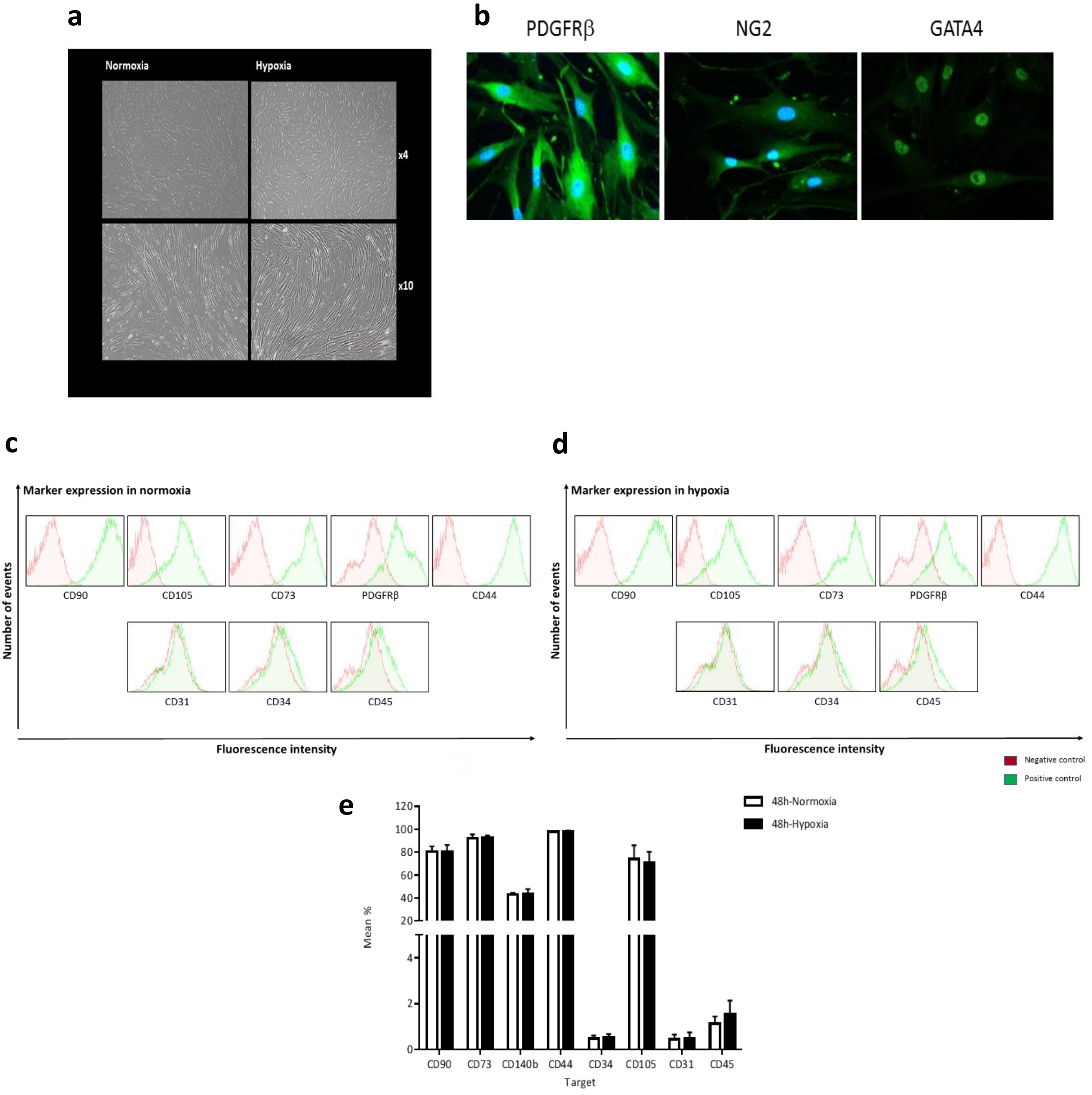

Supplementary Figure II: Ingenuity® Pathway Analysis (IPA) of genes modulated by hypoxia in human APCs, based on two metrics: z-score and *p*-value. A positive or negative z-score value indicates that a function is increased or decreased in hypoxic relative to normoxic cells. In order to enhance the stringency of the analysis, we considered only functions with a z-score > 1 or < -1. The *p*-value (red dots), calculated with the Fischer's exact test, reflects the likelihood that the association between a set of genes in the dataset and a related biological function is significant. Results indicate that *LEP* is implicated in the majority of functions modulated by hypoxia.

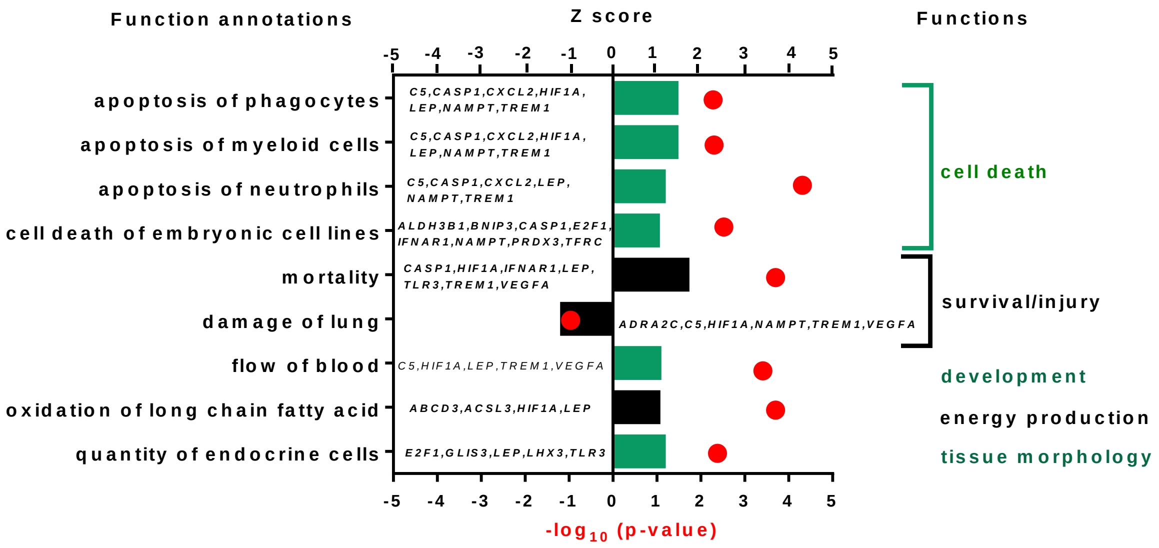

Supplementary Figure III: Effect of miR-210 inhibition on *LEP* mRNA and LEP protein expression and signalling. Experiments were conducted on four cell lines. (a) Bar graph shows validation of effective miR-210 inhibition by the antago-miR. (b-d) Hypoxia-induced upregulation of *LEP* mRNA and LEP protein levels is not affected by miR-210 inhibition (n=4, ****p*<0.001 *vs.* scramble and vehicle, ##*p*<0.01 *vs.* normoxia. (e) Bar graph showing unchanged expression of PTP1B protein under normoxia and hypoxia (n=4, *p*=N.S. *vs.* normoxia. (f) Inhibition of miR-210 upregulates the expression of the target PTP1B protein (n=7, **p*<0.05 *vs.* Scramble). (g) ELISA showed no change in phosphorylated JAK2 levels in response to miR-210 inhibition (n=7, *p*=N.S. *vs.* Scramble).

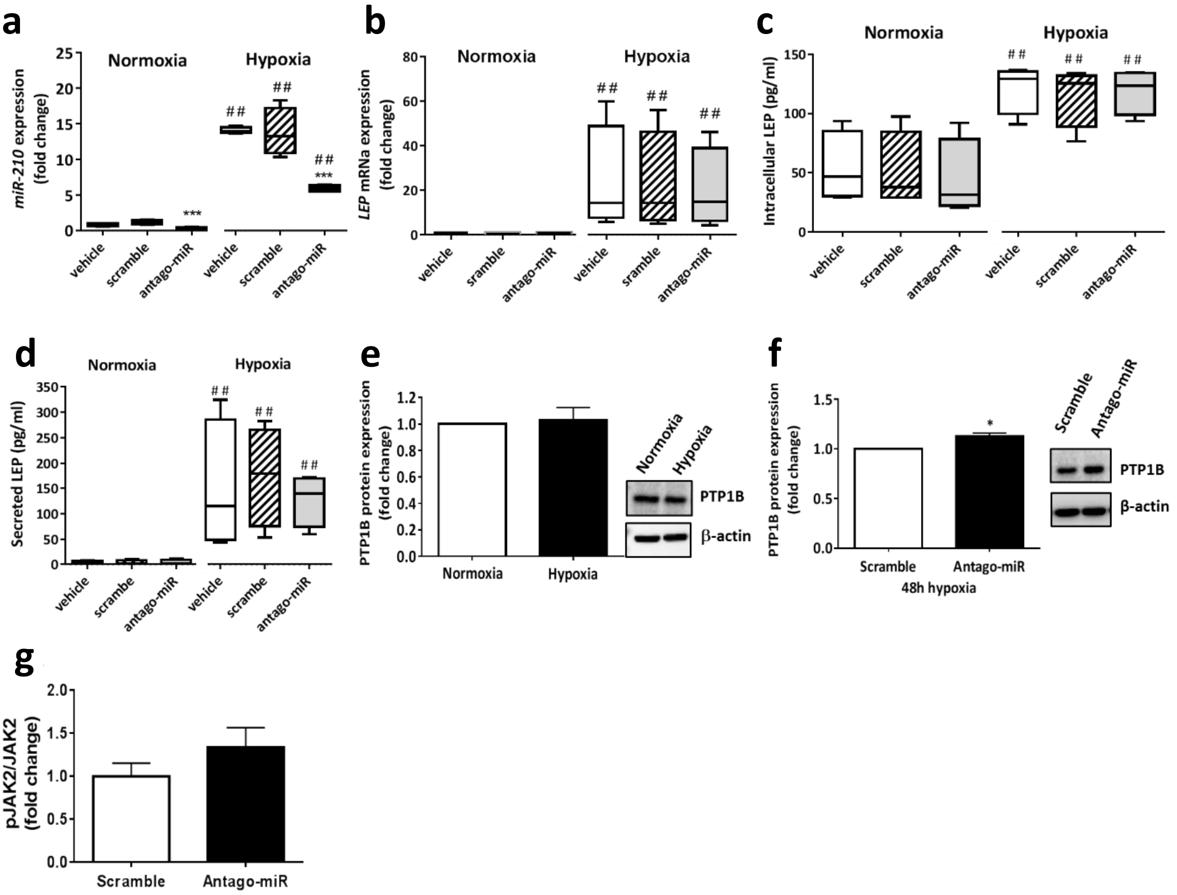

Supplementary Figure IV: Validation of *LEP* and *LEPR* silencing by siRNA. (a) Effective silencing of *LEP* by siRNA ***p<0.001 *vs*. scramble sequence. (b) Hypoxia induces increased leptin secretion, which is inhibited by *LEP* siRNA. *p<0.05 and ***p<0.001 *vs*. normoxia and scramble sequence, respectively. (c and d) Effective *LEPR* silencing in APCs (c, Bar graph shows the average of four biological replicates. ***p<0.001 *vs*. scramble) and HUVECs (d, ***p<0.001 *vs*. scramble). Inserts illustrate representative Western blots of the LEPR protein in APCs and HUVECs transfected with *siLEPR* or scramble.

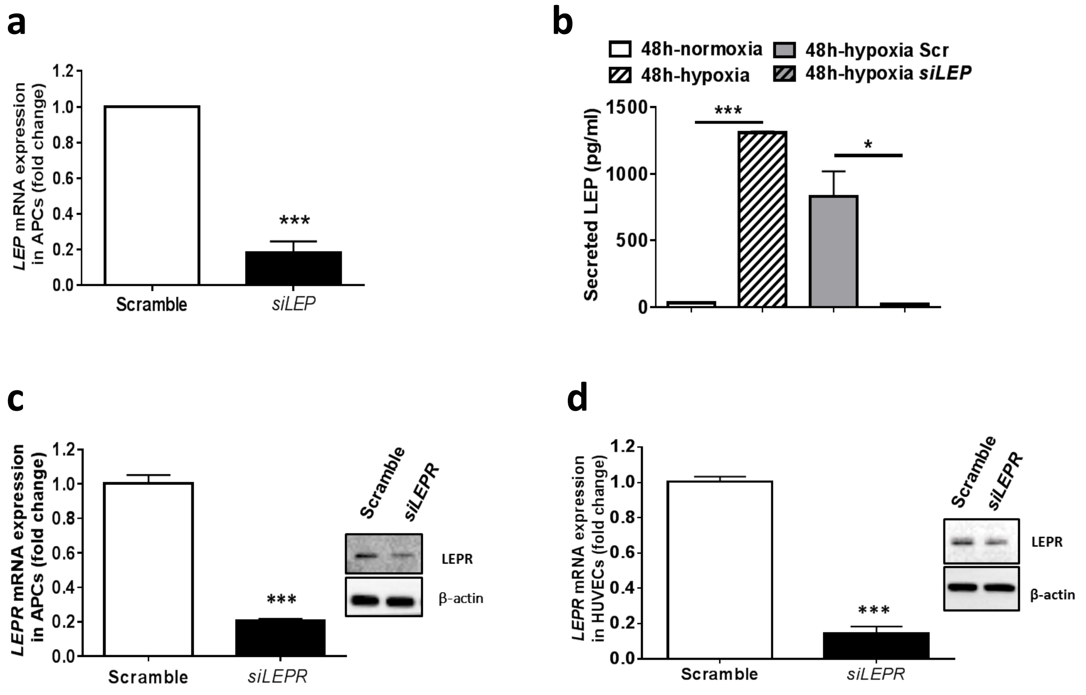

Supplementary Figure V: Original full western blot images displayed in this manuscript and additional information regarding with the immunoblots of phosphorylated and total proteins (Panels 1-6)

Experiments were performed in different APC lines (n ≥ 3) for each control and experimental condition. All experimental conditions were compared with each respective control condition in the same gel. Antibodies used in this manuscript have been previously used in our laboratory as published previously.[1-3] Different titration was performed for the antibodies during the protocol optimization. All PVDF membranes were assessed for the loading control (-actin or occasionally /-tubulin) to confirm that different protein levels were not a result of pipetting error. Membranes were incubated up to 10 min with the ECL prime reagent (GE Healthcare) according to manufacturer's instructions. Only the best and representative images were captured for publication. Image capture was usually performed using ChemiDoc MP system (Bio-Rad) or by Li-cor image 700/800 wavelength (Biosciences). Original Western blotting images were digitalized at 600 dpi by using Image Lab 5.1 software (Bio-Rad). Band densitometry was performed by Image J online software for all bands including targets and loading controls. Ratios among the area calculated for the target and the area calculated for the loading control were assessed in each sample to calculate the relative area of the protein of interest. The area of the control condition was considered 1 and the fold change was calculated for the targeted protein. Using fold change values, we compared the regulatory effect of the experimental condition *vs.* its respective control condition for each APC line at the protein level. After demonstrating no regulation of the selected loading control by the experimental conditions, new gels were often run to achieve representative and improved images for each sample which are included in the final version of this manuscript. Brightness processing were performed if necessary and always applied equally to control and experimental samples. No further improving image procedures were performed.

Panel 1 (refer to text Figure 2e. Hypoxia induces leptin production and secretion by human APCs). Representative original immunoblots performed in a single membrane to confirm LEPR regulation in hypoxic APCs. Bands displayed in this manuscript has been framed into red boxes.

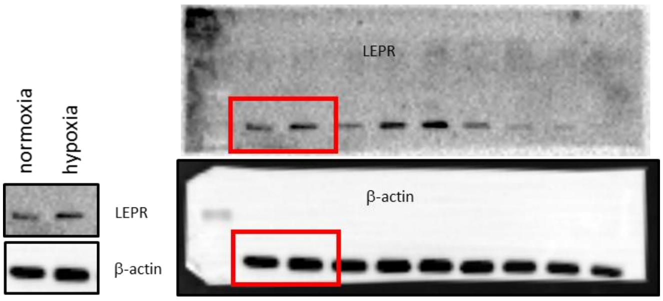

Panel 2 (refer to text Figure 3a. Effect of exogenous rh-leptin on canonical signalling and functional assays in human APCs.). Original images captured using Licor image 700/800 wavelength. Images *i* and *ii* correspond to Li-cor imaging; Image *iii* corresponds to the grey-scale image for image *i*; Image *iv* corresponds to the grey-scale image for image *ii*. Bands displayed in this manuscript has been framed into red boxes.

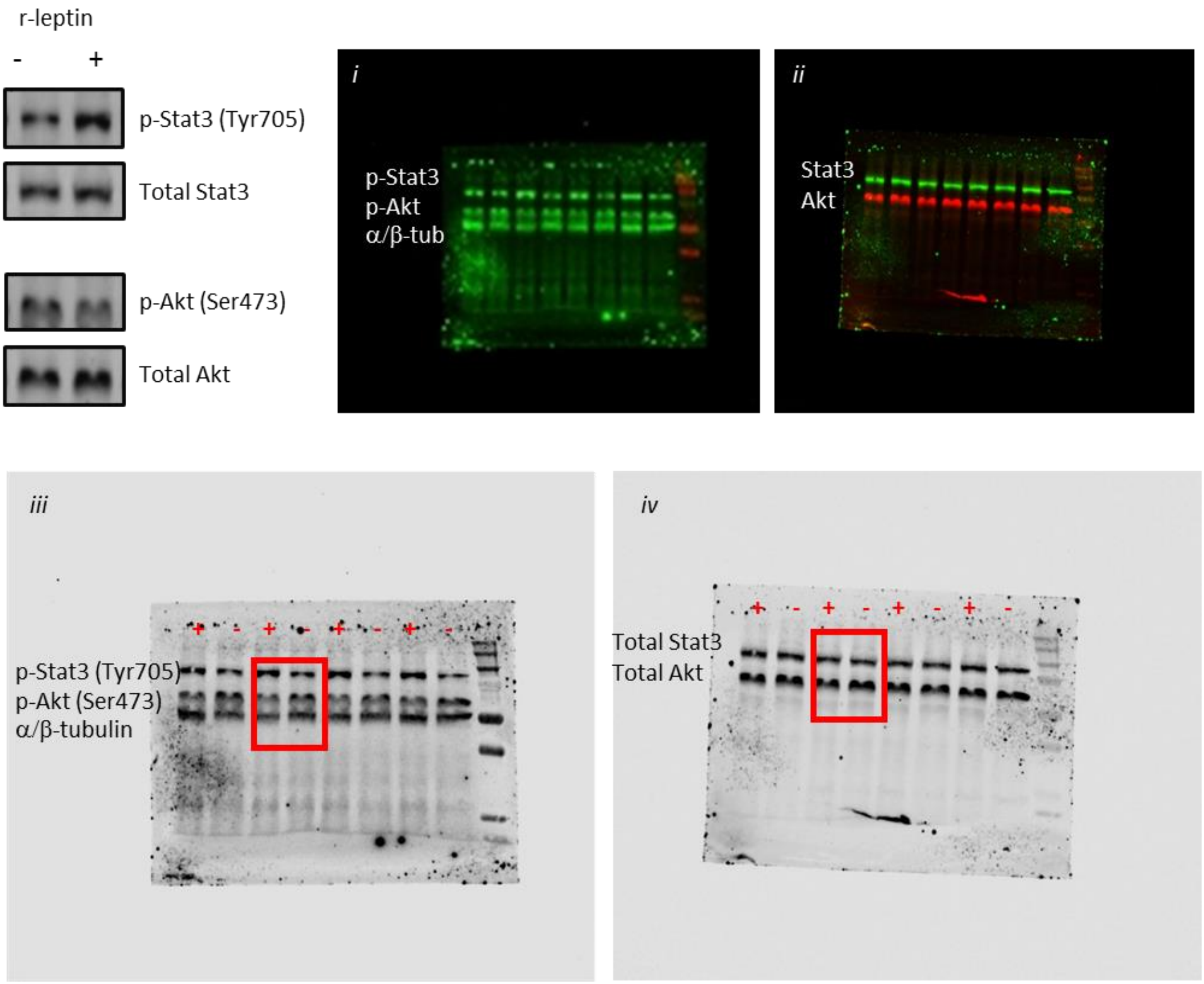

Panel 3 (refer to text Figure 4a. Effect of normoxia and hypoxia on LEP-associated kinases and functional activities of human APCs). Sequential immunoblots performed in a single membrane are indicated in ordinal numbers starting from '1st'. Bands displayed in this manuscript has been framed into red boxes.

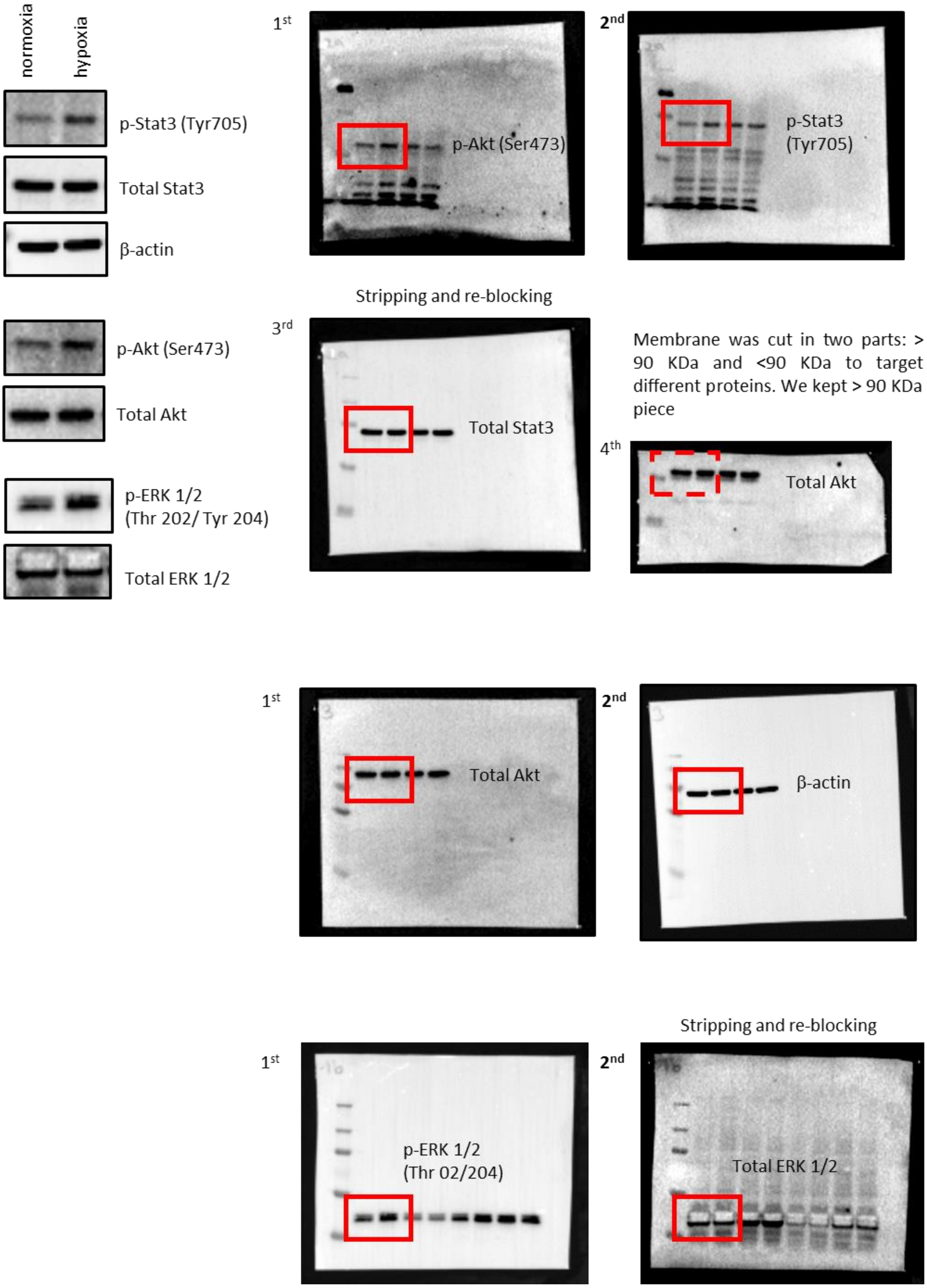

Panel 4 (refer to text Figure 4b. Effect of *LEP* silencing on LEP-associated kinases and functional activities of human APCs). Sequential immunoblots performed in a single membrane are indicated in ordinal numbers starting from '1st'. Bands displayed in this manuscript has been framed into red boxes.

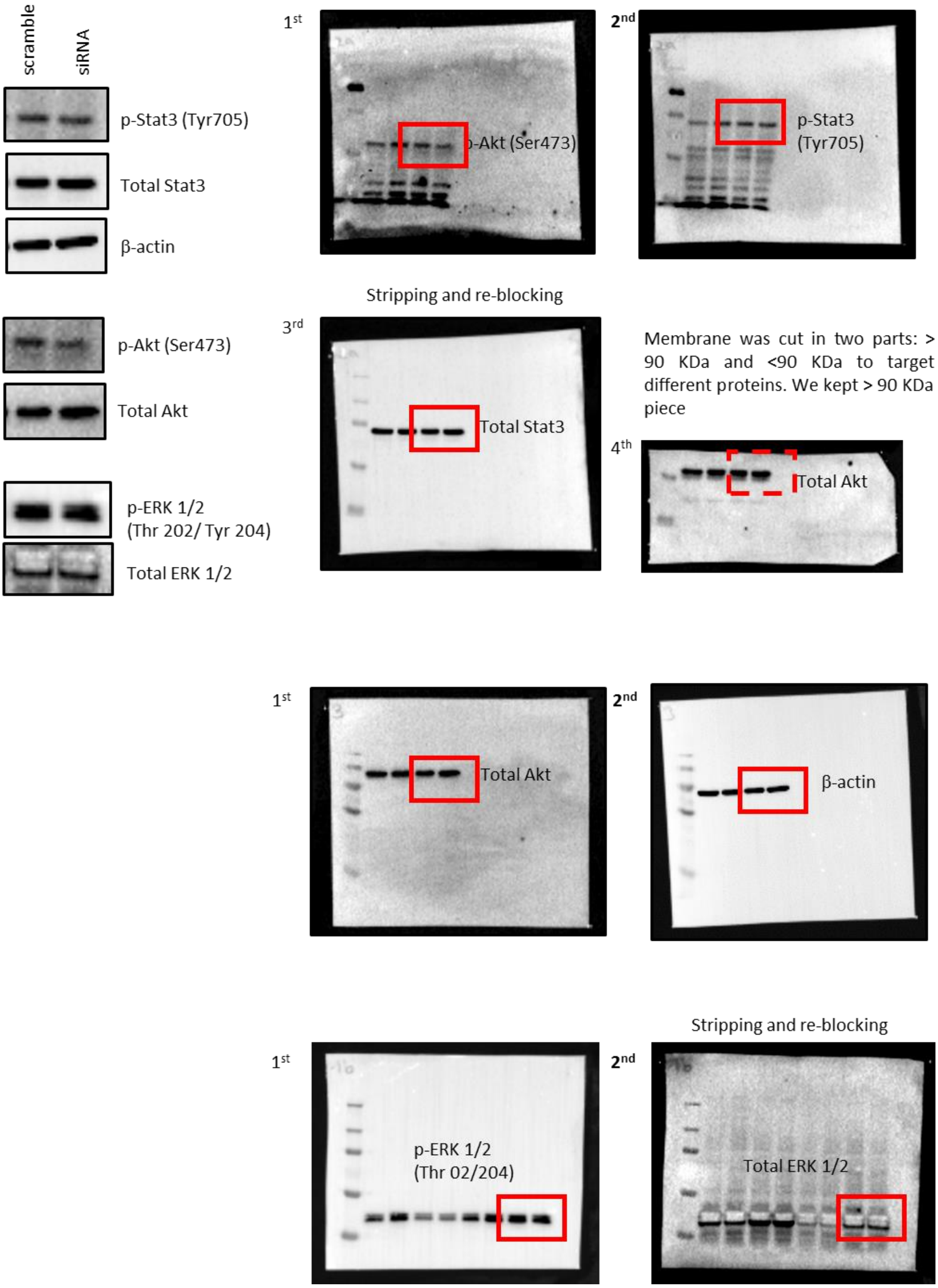

Panel 5 (refer to text Figure 4c. Effect of *LEPR* silencing on LEP-associated kinases and functional activities of human APCs). Sequential immunoblots performed in a single membrane are showed. Bands displayed in this manuscript has been framed into red boxes.

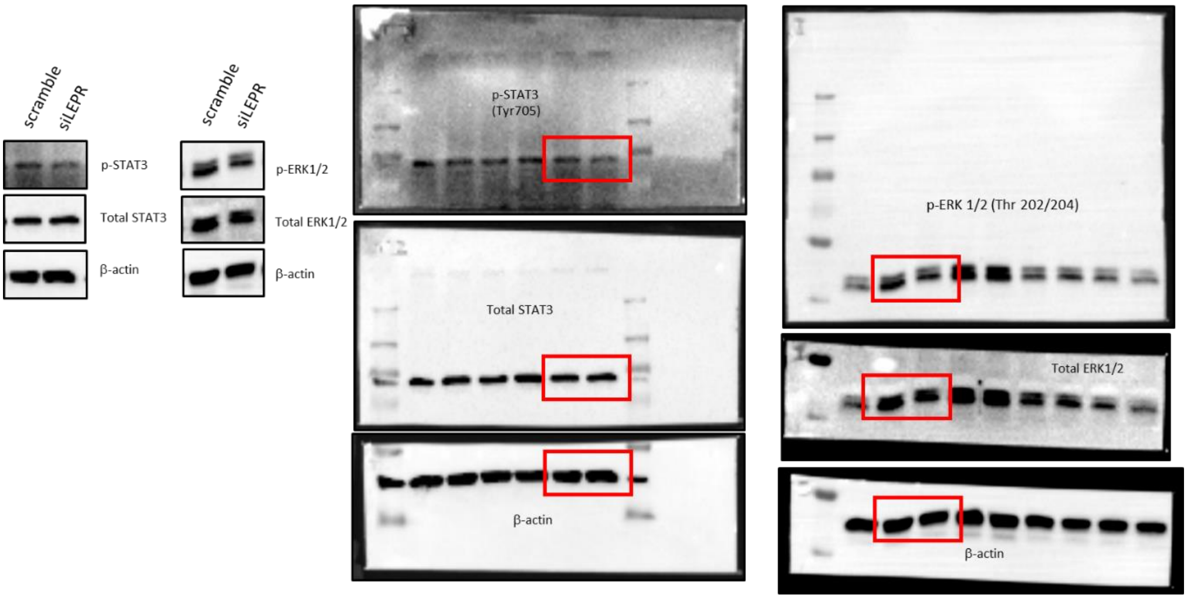

Panel 6 (refer to Supplementary Figure III e. Effect of normoxia and hypoxia on PTP1B protein expression.). Sequential immunoblots (if any) performed in a single membrane are indicated in ordinal numbers starting from '1st'. Bands displayed in this manuscript has been framed into red boxes.

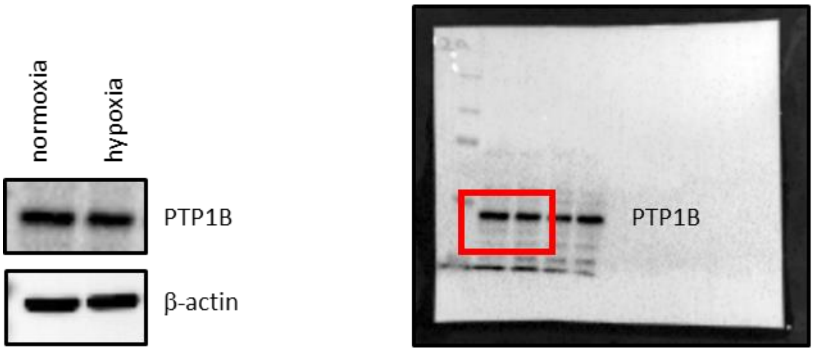

Panel 7 (refer to Supplementary Figure III f. Effect of miR-210 inhibition PTP1B protein expression). Sequential immunoblots (if any) performed in a single membrane are indicated in ordinal numbers starting from '1st'. Bands displayed in this manuscript has been framed into red boxes.

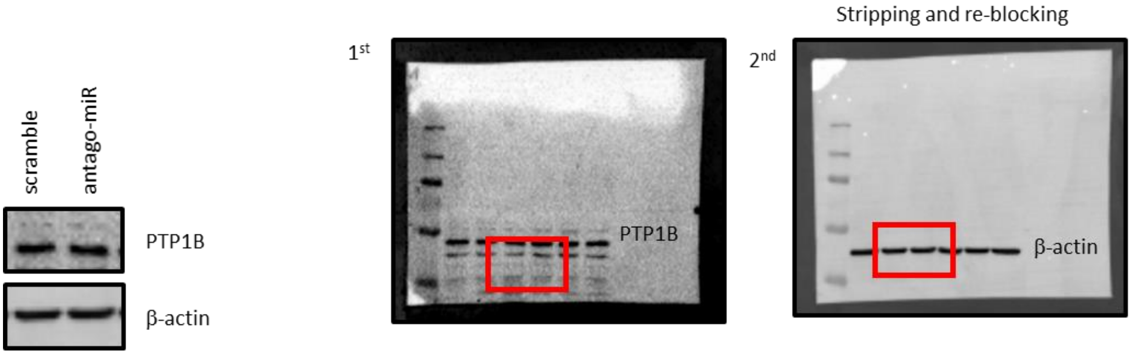

Panel 8 (refer to Supplementary Figure IV c. Validation of *LEP* and *LEPR* silencing by siRNA). Sequential immunoblots performed in a single membrane are showed. *LEPR* silencing in APCs exposed to hypoxia was evidenced by western blot. Bands displayed in this manuscript has been framed into red boxes.

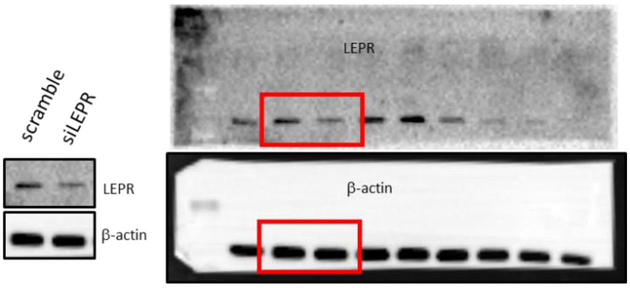

Panel 9 (refer to Supplementary Figure IV d. Validation of *LEP* and *LEPR* silencing by siRNA). Sequential immunoblots performed in a single membrane are showed. *LEPR* silencing in HUVECs exposed to normoxia was evidenced by western blot. Bands displayed in this manuscript has been framed into red boxes.

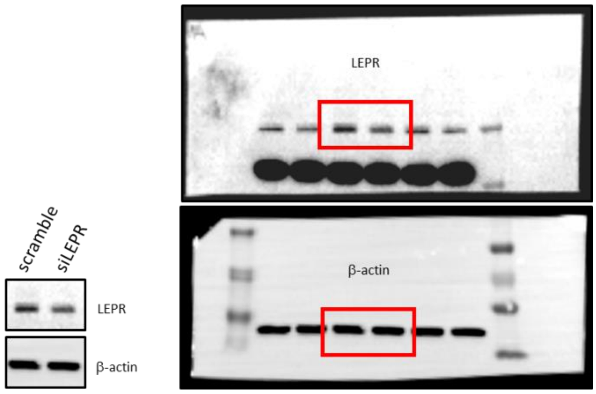

References

1. Katare, R.G. *et al.* Vitamin B1 analog benfotiamine prevents diabetes-induced diastolic dysfunction and heart failure through Akt/Pim-1-mediated survival pathway. *Circulation. Heart failure* **3**, 294-305 (2010).
2. Madeddu, P. *et al.* Phosphoinositide 3-kinase gamma gene knockout impairs postischemic neovascularization and endothelial progenitor cell functions. *Arteriosclerosis, thrombosis, and vascular biology* **28**, 68-76 (2008).
3. Siragusa, M. *et al.* Involvement of phosphoinositide 3-kinase gamma in angiogenesis and healing of experimental myocardial infarction in mice. *Circulation research* **106**, 757-768 (2010).
